# T Cell‐Mediated Transport of Polymer Nanoparticles across the Blood–Brain Barrier

**DOI:** 10.1002/adhm.202001375

**Published:** 2020-11-25

**Authors:** Maxime Ayer, Markus Schuster, Isabelle Gruber, Claudia Blatti, Elisa Kaba, Gaby Enzmann, Olivier Burri, Romain Guiet, Arne Seitz, Britta Engelhardt, Harm‐Anton Klok

**Affiliations:** ^1^ École Polytechnique Fédérale de Lausanne (EPFL) Institut des Matériaux and Institut des Sciences et Ingénierie Chimiques Laboratoire des Polymères Bâtiment MXD Station 12 Lausanne CH‐1015 Switzerland; ^2^ Theodor Kocher Institute University of Bern Freiestrasse 1 Bern CH‐3012 Switzerland; ^3^ École Polytechnique Fédérale de Lausanne (EPFL) Faculté des sciences de la vie Bioimaging and Optics Platform Bâtiment AI, Station 15 Lausanne CH‐1015 Switzerland

**Keywords:** blood–brain barriers, cell‐mediated deliveries, cell‐surface modifications, nanomedicines, nanoparticles

## Abstract

Delivery of therapeutics to the central nervous system (CNS) is challenging due to the presence of the blood–brain barrier (BBB). Amongst various approaches that have been explored to facilitate drug delivery to the CNS, the use of cells that have the intrinsic ability to cross the BBB is relatively unexplored, yet very attractive. This paper presents a first proof‐of‐concept that demonstrates the feasibility of activated effector/memory CD4^+^ helper T cells (CD4^+^ T_EM_ cells) as carriers for the delivery of polymer nanoparticles across the BBB. This study shows that CD4^+^ T_EM_ cells can be decorated with poly(ethylene glycol)‐modified polystyrene nanoparticles using thiol–maleimide coupling chemistry, resulting in the immobilization of ≈105 nanoparticles per cell as determined by confocal microscopy. The ability of these cells to serve as carriers to transport nanoparticles across the BBB is established in vitro and in vivo. Using in vitro BBB models, CD4^+^ T_EM_ cells are found to be able to transport nanoparticles across the BBB both under static conditions as well as under physiological flow. Finally, upon systemic administration, nanoparticle‐modified T cells are shown to enter the brain parenchyma of mice, demonstrating the brain delivery potential of this T cell subset in allogeneic hosts.

## Graphical abstract

CD4^+^ helper T cells can be surface‐modified via thiol–maleimide chemistry with polymer nanoparticles. These modified cells can act as carriers that allow for the transport of the nanoparticle cargo across the blood–brain barrier both in vitro as well as in vivo.

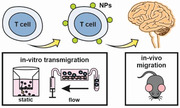

## 1 Introduction

Efficient delivery of drugs across the blood–brain barrier (BBB) is key to the diagnosis and treatment of diseases of the central nervous system (CNS). The BBB is composed of endothelial cells connected by intercellular junctions that prohibit free passage of polar compounds via the paracellular route.^[^ ^1^, ^2^ ^]^ In addition, uncontrolled, transcellular passage of these molecules is hindered by the low pinocytotic activity of the BBB endothelium. Due to the restrictive nature of the BBB, efficient transport to the CNS is essentially limited to lipophilic compounds with molecular weights less than 400 Da.^[^ ^3^ ^]^ This is a major limitation, as a large number of drug candidates to treat brain related diseases do not fulfill these requirements.

Nanoparticles have been extensively investigated to enhance drug delivery across the BBB. Nanoparticle carriers can enhance drug delivery to the CNS in a number of ways: i) by opening tight junctions or inducing local toxic effects that lead to local permeabilization of the BBB; ii) by transcytosis of the nanoparticles across the BBB, iii) by endocytosis of the nanoparticles, followed by intracellular release and exocytosis of their payload; and iv) by a combination of these pathways.^[^ ^4^, ^5^ ^]^

Cell‐based carriers offer unique opportunities to improve targeted delivery of nanoparticles, to enhance their blood circulation times and to facilitate transport of nanoparticles across challenging physiological barriers.^[^ ^6^, ^7^, ^8^ ^]^ While cell‐mediated delivery of nanoparticles has been explored in a number of cases to combat cancer, only relatively little effort has been made to use this concept to enhance drug delivery to the CNS.

There is a number of cell types that possess the innate ability to cross the BBB and thus are potentially attractive carriers to facilitate drug delivery to the CNS.^[^ ^9^, ^10^ ^]^ One example are human peripheral monocyte‐derived macrophages and mouse bone marrow‐derived macrophages. These cells readily internalize their payload via phagocytosis. A potential caveat of this approach is that it is the monocytes rather than differentiated macrophages that naturally circulate in the blood‐stream. In a series of reports, however, Kabanov and coworkers convincingly demonstrated that bone marrow‐derived macrophages can deliver catalase containing nanoparticles in order to attenuate neuroinflammation and nigrostriatal regeneration in Parkinson's disease.^[^ ^11^, ^12^, ^13^, ^14^, ^15^ ^]^ While macrophages may cross the BBB under CNS inflammatory conditions, the observation that myeloid cells present in the CNS are long lived and not frequently replaced by monocyte‐derived myeloid cells^[^ ^16^ ^]^ makes this cell subset less attractive for targeting the CNS under non‐inflammatory conditions. Additional challenges associated with the use of macrophages are: i) the payload is localized intracellularly, which adds another barrier to the overall drug delivery process; and ii) macrophages once in a tissue entertain inflammatory responses. In another recent conceptually related contribution, neutrophils loaded with liposomes that contained paclitaxel were successfully used to suppress postoperative glioma recurrence.^[^ ^17^ ^]^ While this approach enabled to slow recurrent tumor growth in a mouse glioma model, the use of neutrophil‐based carriers, similar to macrophages, is essentially limited to pathologies that are accompanied by inflammation.^[^ ^18^, ^19^ ^]^ Neural stem cells (NSCs) are another cell subset that are attractive potential carriers to mediate nanoparticle delivery to the CNS. NSCs have also been reported to possess tumor tropic properties and to be able to target intracranial gliomas when administered intravenously.^[^ ^20^ ^]^ NSCs, which were modified with 800 nm diameter polystyrene particles and administered via the tail vein of glioma‐bearing mice, were shown to be able to transport the nanoparticle payload to the tumor site.^[^ ^20^ ^]^ NSCs, however, were not found in the brain after systemic injection of the cells in mice without intracerebral glioma,^[^ ^21^ ^]^ which supports the notion that NSCs need inflammatory cues for crossing the BBB and would not be a suitable cellular carrier to reach non‐inflamed areas of the CNS.

**Figure 1:**
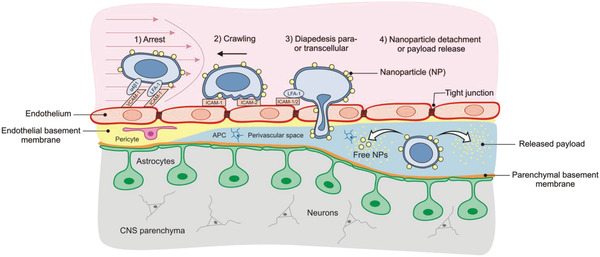
Schematic illustration of CD4^+^T_EM_cell‐mediated transfer of nanoparticles across the BBB. The cartoon shows the putative transfer of nanoparticles conjugated to the cell surface across the BBB endothelium, followed by nanoparticle detachment and drug release upon arrival in the CNS (APC = antigen presenting cell, NPs = nanoparticles).

Activated effector/memory CD4^+^ helper T cells (CD4^+^ T_EM_ cells) are another attractive, yet unexplored, class of circulating immune cells that possess great potential to enhance drug delivery to the CNS. While naïve T cells recirculate between the lymphoid organs, activated CD4^+^ T_EM_ cells are reprogrammed such that they can travel to non‐lymphoid organs including the CNS also in the absence of neuroinflammation.^[^ ^22^, ^23^ ^]^ The migration of CD4^+^ T_EM_ cells across the BBB is a multistep process and is schematically illustrated in **Figure** **1**. The sequence of events involves T‐cell rolling and arrest on BBB endothelium, followed by T‐cell crawling, which is mediated by the endothelial adhesion molecules, intercellular adhesion molecule‐1 (ICAM‐1) and intercellular adhesion molecule‐2 (ICAM‐2), eventually leading to T‐cell migration across the BBB at a junctional or non‐junctional site permissive for diapedesis.^[^ ^24^, ^25^ ^]^ CD4^+^ T_EM_ cells possess a number of characteristics that make them promising drug delivery carriers to the CNS. Foremost, activated CD4^+^ T_EM_ cells cross the BBB independent of their antigen specificity and can thus be explored in an allogeneic context, omitting the necessity to isolate and differentiate these cells from the patient proper.^[^ ^26^ ^]^ They also possess the ability to cross the BBB in the absence of neuroinflammation making them suitable for CNS drug delivery for diagnostic purposes or in neurodegenerative disorders.^[^ ^22^ ^]^ Furthermore, CD4^+^ T_EM_ cells, in contrast to effector/memory CD8^+^ cytotoxic T cells, which may share similar trafficking characteristics, lack the intrinsic potential to kill target cells, which is an asset in terms of their safe use as drug delivery carriers. Another attractive characteristic of CD4^+^ T_EM_ cells is that their ability to cross the BBB is not limited by the immune privileged nature of the CNS. Activated CD4^+^ T_EM_ cells cross the BBB independent of the antigen specificity. If these T cells do not recognize their cognate antigen on perivascular or leptomeningeal antigen presenting cells (APCs), then they will not cross the glia limitans but undergo apoptosis and be drained from the CNS.^[^ ^27^ ^]^ Recognition of a cognate antigen on the perivascular or leptomeningeal APCs, however, can induce local T cell inflammation in CNS perivascular spaces around post‐capillary venules or in the leptomeningeal spaces ultimately paving the way for the immune cells to cross the glia limitans and infiltrate the CNS parenchyma.^[^ ^27^, ^28^ ^]^ This is interesting as it allows, depending on the activation state of the T cells, to deliver to the perivascular space, and from there rely on diffusion of the cargo across the glia limitans, or to shuttle nanoparticle‐decorated T cells directly into the CNS.

This article demonstrates the use of CD4^+^ T_EM_ cells to enhance the delivery of nanoparticles to the CNS. In this report, we explore the feasibility of CD4^+^ T_EM_ cells to facilitate transport of 200 nm diameter, poly(ethylene glycol) (PEG)‐modified polystyrene model nanoparticles across the BBB. The PEG chains that constitute the corona of the nanoparticles were functionalized with maleimide groups to allow covalent conjugation of the nanoparticles to thiol groups present on the cell surface. The localization and distribution of the nanoparticles on the cell surface was assessed through confocal microscopy experiments. Cell‐surface coupling of the nanoparticles was not found to induce necrosis or apoptosis and also did not alter the ability of the T cells to bind to recombinant ICAM‐1. Nanoparticle‐decorated T cells were then investigated for their ability to migrate across a mouse BBB model in vitro both under static conditions in a transendothelial migration assay as well as under physiological flow. Finally, these T cells were utilized to deliver nanoparticles to the CNS in mice demonstrating the brain delivery potential of these T cell conjugates under allogeneic conditions.

## 2 Results and Discussion

### 2.1 Nanoparticle Synthesis and T Cell‐Surface Modification

**Figure 2:**
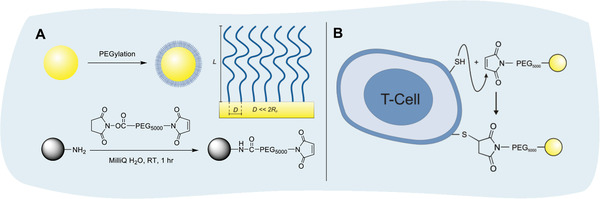
A) Synthesis of maleimide‐functionalized PEGylated polystyrene nanoparticles. B) Coupling of maleimide‐functionalized PEGylated polystyrene nanoparticles to thiol groups present on the T cell surface. For simplification, only one PEG chain per nanoparticle is shown.

To explore the feasibility of CD4^+^ T_EM_ cells to facilitate transport of therapeutic cargo across the BBB, this study used model nanoparticles, which were obtained by surface modification of 200 nm diameter, fluorescently labeled (BODIPY) amine functionalized polystyrene beads. These particles were modified with a heterobifunctional PEG derivative to introduce maleimide groups, which were subsequently used to conjugate the nanoparticles to thiol groups of cell‐surface proteins (**Figure** **2**). Further details on the characterization of the nanoparticles are provided in Figures S1–S3, Supporting Information. As they present a large number of maleimide groups, coupling of the nanoparticles to thiol groups on the cell surface is most likely to result in multipoint attachment. Control nanoparticles with a methoxy‐PEG corona were produced in the same way as their maleimide‐functionalized counterparts.

Cell‐surface conjugation of the maleimide‐functionalized polystyrene nanoparticles was performed as shown in Figure 2B following a strategy reported earlier by Irvine and coworkers for the coupling of liposomes to the surface of B cells, T cells, and hematopoietic stem cells.^[^ ^29^ ^]^ The T cells investigated in this study were modified by reacting with nanoparticles at a concentration of 5000 nanoparticles/cell. As a control experiment, cells were exposed to methoxy‐functionalized PEGylated polystyrene nanoparticles. Surface modification of the T cells with the nanoparticles was followed using flow cytometry (Figure S4, Supporting Information). While these experiments indicated that non‐specific binding of the control particles also takes place, the use of the maleimide‐functionalized particles resulted in a >100‐fold increase in fluorescence intensity illustrating the successful covalent coupling of the maleimide‐functionalized polystyrene nanoparticles to the cell‐surface thiol groups.

### 2.2 Characterization of Nanoparticle‐Modified T Cells

**Figure 3:**
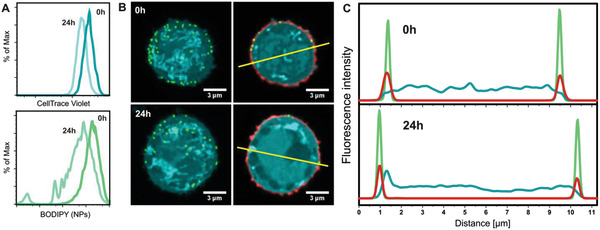
A) Flow cytometry measurements directly after T‐cell modification and 24 h later. Top view shows a histogram of scatter gated live T cells stained with CellTrace Violet to observe cell division over 24 h. Bottom view shows a histogram of scatter gated live cells for nanoparticle retention assessed using BODIPY labeled fluorescent nanoparticles. B) left: Maximum intensity projections of nanoparticle (green) decorated T cells (cyan). T cells were stained with CellTrace Violet. Right: cross‐sectional confocal image of a T cell with an additional membrane WGA‐Texas Red‐X (red) staining. C) Fluorescence intensity profiles (yellow line in B) directly after modification and 24 h later showing the overlapping of nanoparticle‐associated fluorescence (green) and the membrane stain (red) on the edge of the whole cell stain (cyan).

The concentration, localization, and distribution of the nanoparticles on the cell surface directly after cell‐surface attachment as well as after 24 h were studied by flow cytometry and confocal microscopy. For the flow cytometry experiments, prior to cell‐surface conjugation, cells were stained with a proliferation marker (CellTrace Violet), which allowed to simultaneously monitor the T cell proliferation and nanoparticle cell‐surface attachment. The flow cytometry results, which are presented in **Figure** **3**A, show that after 24 h the cells have gone through one cycle of cell division. Monitoring the nanoparticle‐associated (BODIPY) fluorescence by flow cytometry revealed that nanoparticles remained attached during cell division. One cycle of cell division, however, was accompanied by approximately a fourfold decrease in the BODIPY (nanoparticle‐associated) fluorescence, which could be indicative of a partial loss of surface‐attached nanoparticles. In addition, a significant broadening of the nanoparticle‐associated fluorescence distribution is observed upon cell division. This indicates an uneven repartition of the particles on the daughter cells, which could be the consequence of a heterogeneous distribution of the nanoparticles on the initial cell surface (vide infra).

To determine the number and distribution of nanoparticles on the cell surface, cells were treated with CellTrace Violet to visualize the cell body and with WGA‐Texas Red‐X to stain the cell membrane, and investigated by confocal microscopy. Figure 3B shows maximum intensity projections of nanoparticle‐modified T cells as well as 2D images of a cross‐sectional plane of these cells directly after modification and 24 h later. Figure 3C presents fluorescence intensity profiles that are obtained from analyzing the 2D cross‐sectional images in Figure 3B along the yellow lines. The images in Figure 3B and the intensity profiles in Figure 3C clearly indicate that the nanoparticles co‐localize with the cell membrane and that the position of the nanoparticles does not significantly change over a period of 24 h. It is also evident from Figure 3B that the nanoparticles are non‐homogeneously distributed over the cell surface. To determine the average number of nanoparticles per cell, 3D reconstructions of nine cells were analyzed, both directly after cell‐surface modification as well as after 24 h. On average, 105 ± 39 nanoparticles/cell were counted directly after modification, which corresponds to an estimated 1% of the total available surface area of a cell. After 24 h, 34 ± 18 nanoparticles/cell were counted, which represents approximately threefold decrease in the number of particles per cell compared to directly after the cell‐surface modification. Based on the total increase in available surface area upon one cycle of cell division, a twofold decrease would be expected. This finding suggests a partial loss of the surface bound cargo upon cell division and is in agreement with the flow cytometry results presented in Figure 3A.

**Figure 4:**
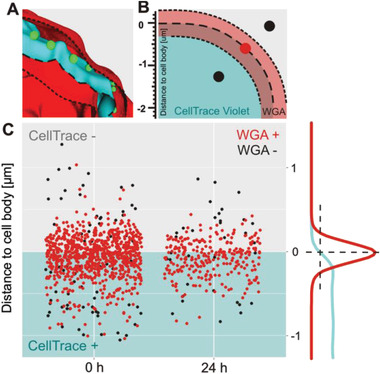
A) Surface generated from image processing (Imaris software: Bitplane, Oxford Instrument) showing the edge of CellTrace Violet (cyan) whole cell staining (‒ ‒ ‒ ‒ ‒), inner and outer boundaries of the WGA‐Texas Red‐X (red) membrane staining (˗ ˗ ˗ ˗ ˗) and 200 nm nanoparticles (green). B) Schematic explanation for the nanoparticle distribution observed in (C) around the cell edge. C) Statistical distribution of nanoparticles with respect to their distance to the whole cell edge (CellTrace Violet). Each dot represents a single nanoparticle. Red dots represent nanoparticles which are found between the inner and outer boundaries outlined by the membrane staining (WGA‐Texas Red‐X) and black dots those which are not. Right: typical fluorescence intensity profiles for the CellTrace Violet and WGA‐Texas Red‐X channels around the cell edge.

Next, the confocal microscopy images were used to analyze the position and distribution of the nanoparticles. The image analysis protocol is schematically illustrated in **Figure** **4**A,B and involves measuring the distance between the nanoparticle (green) and the perimeter of the cell, which was taken as the edge of the CellTrace Violet stain (cyan), as well as calculating the percentage of nanoparticles that are found between the inner and outer boundaries generated by the WGA‐Texas Red‐X membrane stain (red). Figure 4C summarizes the results of analyzing a total of nine cells both at *t* = 0 and 24 h. This figure presents, for each nanoparticle, the distance between its position and the cell edge, which is defined as 0 µm. Each dot represents a single nanoparticle. A red dot represents a nanoparticle that is found within the boundaries of the membrane stain. Black dots represent nanoparticles that are located outside the boundaries defined by the membrane stain. Directly after surface modification, the nanoparticles are localized at a distance of ≈−66 ± 305 nm from the edge of the cell. A very similar number is measured after 24 h, that is, −65 ± 256 nm. Approximately 90% of the nanoparticles were found to co‐localize with the WGA‐Texas Red‐X membrane stain, both directly after cell‐surface modification and after 24 h. The confocal microscopy experiments indicate that the nanoparticles are located at the cell surface and remain there at least for a period of 24 h. Not surprising given the non‐phagocytic nature of the CD4^+^ T_EM_ cells, during the time course of these experiments there was no indication for nanoparticle internalization by the cells.

### 2.3 Viability and In Vitro Functional Analysis of Nanoparticle‐Decorated T Cells

**Figure 5:**
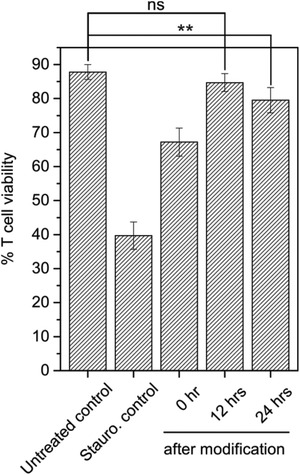
T‐cell viability assay: untreated control T cells and staurosporine treated control T cells (24 h). Then, nanoparticle functionalized T cells directly after modification, 12, and 24 h after modification. The results are shown for two independent experiments performed in triplicate. Error bars on the histograms represent standard deviation.*p*‐values were determined by *t*‐test (ns:*p* > 0.05 **p < 0.01 is).

To investigate the possible effect of cell‐surface modification on the viability of the T cells, nanoparticle‐decorated T cells as well as unmodified T cells were studied using an Annexin V–DAPI apoptosis/necrosis assay. T‐cell viability was assessed directly after surface modification as well as at 12 and 24 h. As a positive control for apoptosis, T cells were treated with staurosporine. The flow cytometry results of these experiments are provided in Figure S5, Supporting Information, and **Figure** **5** presents the T‐cell viabilities as obtained from flow cytometry. The results in Figure 5 show that while there is an initial decrease in viability directly after cell‐surface modification, the cells recover as time progresses. The initial decrease is viability is attributed to an osmotic shock that the cells experience as the nanoparticle immobilization is performed in a 1/1 v/v DPBS/MilliQ water medium. The percentage of viable cells increased from ≈67% directly after T cell‐surface modification to >80% after 12 h and longer.

**Figure 6:**
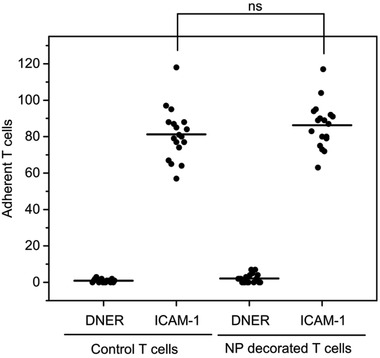
Cell count of a binding assay on recombinant ICAM‐1 and DNER coated wells for unmodified control cells and nanoparticle (NP)‐decorated T cells performed at room temperature for 30 min under moderate shear conditions. Each dot represents one cell count from the diagonal of a 10 mm x 10 mm/10 divisions counting reticle using a 20x objective. The figure represents the results of two independent assays performed in triplicate. Each well (i.e., replicate) was counted at three different positions. The horizontal bar represents the mean over all counts.*p*‐value was determined by *t*‐test (ns:*p* > 0.05).

The functional properties of the nanoparticle‐decorated CD4^+^ T_EM_ cells were investigated in a number of different experiments. First, the ability of the surface‐modified T cells to bind to immobilized recombinant ICAM‐1 was assessed. ICAM‐1 has been identified as a critical adhesion molecule that mediates T‐cell polarization and crawling prior to diapedesis of CD4^+^ T_EM_ cells across the BBB.^[^ ^25^, ^30^ ^]^ For this experiment, ICAM‐1 was coated on a PTFE diagnosis slide and unmodified control T cells or nanoparticle‐carrying T cells were incubated under moderate shear. As a control experiment, T cells were exposed to slides that presented delta/notch‐like epidermal growth factor (DNER), which is a protein that is irrelevant to the diapedesis of CD4^+^ T_EM_ cells. **Figure** **6** plots the number of cells counted per field of view upon analysis of the slides with a counting reticle. The results in Figure 6 demonstrate that the presence of ≈105 polystyrene nanoparticles on the cell surface does not compromise the ability of the CD4^+^ T_EM_ cells to bind to recombinant ICAM‐1. The total surface area covered by the nanoparticles can be estimated to be less than 1%. The absence of adherent cells on the DNER coated wells indicates that binding of the T cells to recombinant ICAM‐1 is a specific ligand (LFA‐1) mediated process.

**Figure 7:**
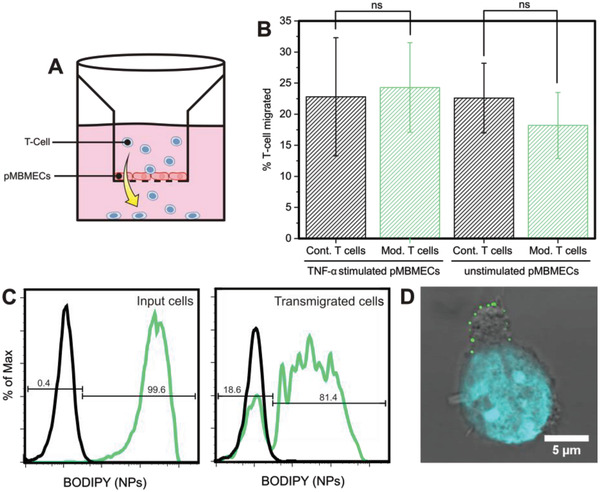
A) Schematic overview of the two‐chamber in vitro transendothelial migration assay (TEM). B) Percentage of migrated T cells across TNF‐*α*stimulated and unstimulated pMBMEC monolayers: comparison between unmodified control (Cont.) T cells and nanoparticle‐decorated T cells (Mod.).*p*‐values were determined by*t*‐test (ns:*p* > 0.05). The histogram presents the results of two independent experiments performed in triplicate and the error bars are standard deviations. C) One representative flow cytometry analysis showing histograms of nanoparticle‐associated fluorescence of live scatter gated T cells. (left) T cell input for TEM assay across a TNF‐*α*stimulated pMBMEC monolayer and (right) migrated T cells. Black: control unmodified T cells; green: nanoparticle functionalized T cells. D) Confocal micrograph of a nanoparticle (green) functionalized T cell (transmission image, nucleus stained with DAPI), which migrated across a TNF‐*α*stimulated pMBMEC monolayer. Nanoparticles are visible at the trailing edge (uropod) of the T cell.

In a second experiment, the question whether nanoparticle‐decorated CD4^+^ T_EM_ cells retain their ability to cross the BBB was assessed in vitro. To this end, a transendothelial migration assay across a primary mouse brain microvascular endothelial cell (pMBMEC) monolayer was performed under static conditions (**Figure** **7**A). This model retains BBB characteristics in vitro such as complex tight junctions and low permeability.^[^ ^31^ ^]^ The assay was performed on both TNF‐*α* stimulated as well as unstimulated pMBMEC monolayers for 6 h. TNF‐*α* stimulation of endothelial cells increases the expression of ICAM‐1 on the surface of the barrier mimicking an inflamed BBB, which is typically found in many neuroinflammatory related disorders. Flow cytometry analysis revealed that 24 ± 7% of nanoparticle‐modified T cells migrated across the TNF‐*α* stimulated and 18 ± 5% across the unstimulated pMBMEC monolayer (Figure 7B). These percentages were not significantly different from those measured for unmodified control T cells migrating across TNF‐*α* stimulated and unstimulated pMBMEC monolayers under the same static conditions, which were 23 ± 10% and 23 ± 6%, respectively. As can be seen from the flow cytometry histograms in Figure 7C, the majority of the T cells were able to transport nanoparticles across the pMBMEC monolayer. Flow cytometry analysis revealed that on average 79 ± 2% of the nanoparticle‐modified CD4^+^ T_EM_ cells were able to transport part of their initial cargo across the pMBMEC monolayer (Figure 7C). Note that Figure 7C shows flow cytometry data from a single experiment, whereas the 79 ± 2% refers to the average over two experiments that were performed in triplicate. Comparison of the flow cytometry results of the input and the transmigrated nanoparticle‐modified T cells shows a decrease in nanoparticle‐associated fluorescence for the nanoparticle‐decorated CD4^+^ T_EM_ cells that migrated across the pMBMEC monolayer. This decrease, which was similar for the non‐stimulated (9‐fold) and TNF‐*α* stimulated monolayer (13‐fold), indicates a partial loss of the nanoparticle payload and may be attributed to shear forces that the T cells are exposed to on crossing the pMBMEC monolayer, clustering of nanoparticles at the uropod during T‐cell polarization and migration, as well as the possible loss of integrins from the T cell surface to which nanoparticles are tethered. Nanoparticles that are lost from the T cell surface during transmigration remain associated with the pMBMEC monolayer as observed by epifluorescence microscopy (Figure S6, Supporting Information). Confocal images of nanoparticle‐modified T cells after transmigration showed nanoparticle clustering at the uropod. Nanoparticle accumulation at the uropod (Figure 7D) has also been reported elsewhere during migration of lipid nanoparticle conjugated CD8^+^ effector T cells on an endothelial cell monolayer toward a chemoattractant^[^ ^32^ ^]^ and was attributed to membrane protein reorganization during T‐cell polarization.^[^ ^33^ ^]^

**Figure 8:**
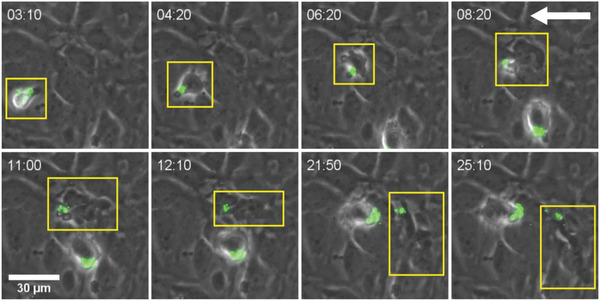
Live cell imaging of a nanoparticle (green) decorated T cell (yellow rectangle) arresting (image at [03:10]), crawling against the flow (images at [04:20] and [06:20]), crossing a pMBMEC monolayer (images from [08:20] to [12:10]), and finally crawling below the pMBMEC layer (images at [21:50] and [25:10]). The white arrow (top right corner) indicates the direction of the flow. Time is indicated in left corners of each images as [min:sec].

Next, the migration of nanoparticle‐decorated CD4^+^ T_EM_ cells across an in vitro BBB model under physiological flow was investigated.^[^ ^34^ ^]^ The same TNF‐*α* stimulated endothelial BBB model as described above was used. The behavior of nanoparticle‐modified T cells was compared to that of unmodified, control T cells with time‐lapse live cell imaging using differential interference contrast microscopy to detect both the T cells and the pMBMECs, and epifluorescence microscopy to detect the nanoparticles (green). T cells were allowed to initiate interaction with the pMBMEC monolayer at a reduced flow rate corresponding to a wall shear stress of 0.2 dyne cm^−2^ during an accumulation period of 5 min. The flow rate was then increased to generate a wall shear stress of 1.5 dyne cm^−2^ to mimic the physiological flow conditions typically encountered within CNS post‐capillary venules. **Figure** **8** shows a sequence of eight images that illustrates the multistep extravasation of a nanoparticle‐decorated CD4^+^ T_EM_ cell across the BBB (video is available as Movie_Figure S8, Supporting Information). First, a nanoparticle‐modified T cell adheres to the endothelial surface during the accumulation phase (image time 03:10 [min:sec]). At this stage, the T cell is surrounded by a halo of light showing that it interacts with the luminal side of the pMBMEC monolayer and the nanoparticles (green) are visible on the T cell. Following attachment and polarization, the T cell crawls and nanoparticles cluster at the uropod as visible in the image taken at 04:20. The T cell then was observed to further crawl against the direction of flow to eventually reach a site on the pMBMEC monolayer permissive for diapedesis (image time 06:20). Then, the nanoparticle‐modified T cell can be seen during extravasation, partially through the pMBMEC monolayer in the two images recorded at 08:20 and 11:00. Only the uropod with a nanoparticle cluster remains above the pMBMEC monolayer at this stage. The largest part of the T cell has crossed the pMBMEC monolayer and its dark protrusions are visible below the endothelial cells. The extravasation of the nanoparticle‐modified T cells takes ≈6 min in total from the point that the T cell has found a site permissive for diapedesis until the entire cell is found below the pMBMEC monolayer (image at 12.10). Once the T cell is under the pMBMEC monolayer, it continues to crawl below carrying its nanoparticulate cargo along (images at 21:50 and 25:10). Toward the end of the sequence, some nanoparticles are released from the T cell surface.

**Figure 9:**
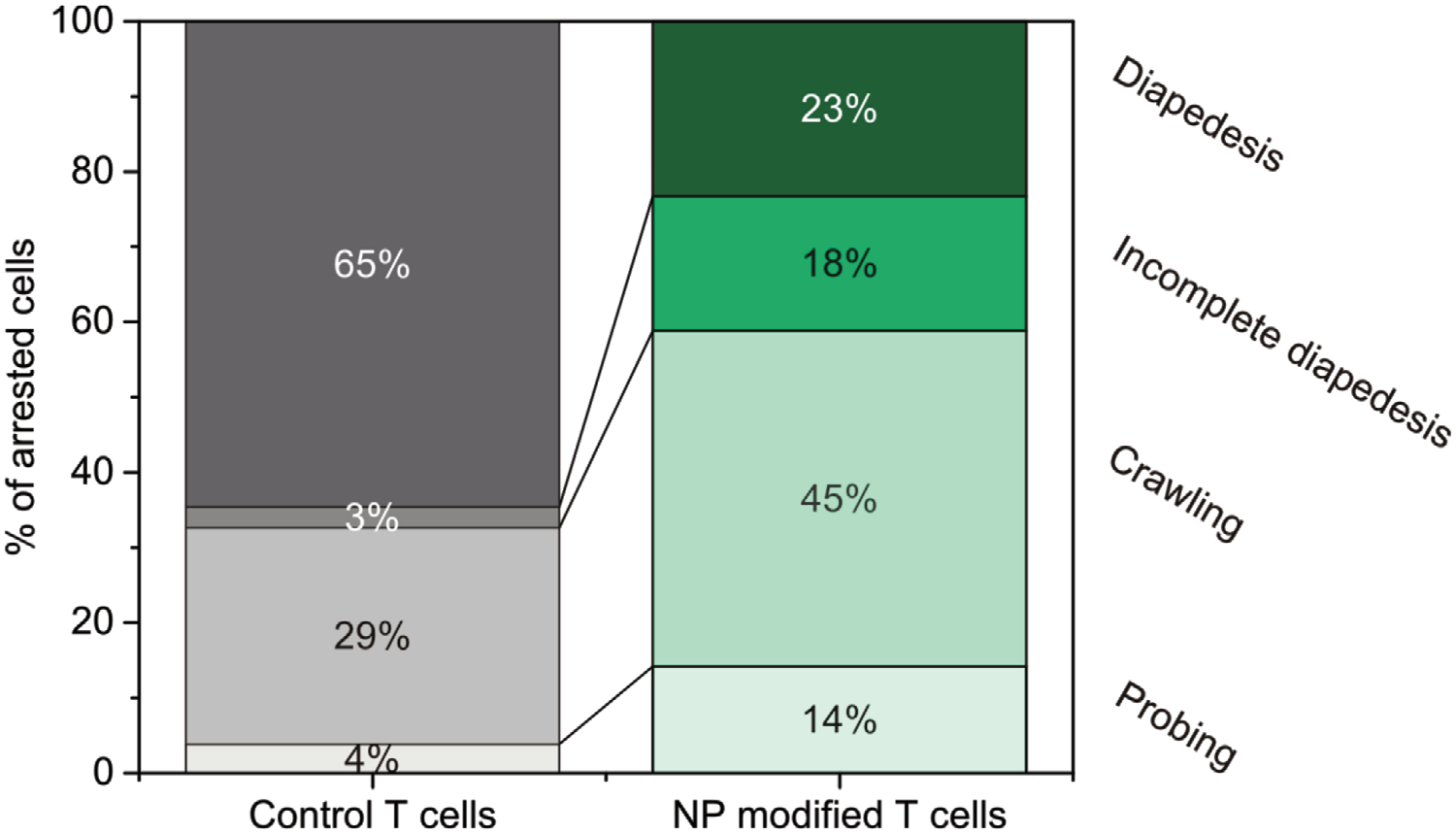
Percentage of arrested, unmodified (left), or nanoparticle‐modified (right) T cells that undergo successful diapedesis, incomplete diapedesis, or which remain in a crawling or probing state on the pMBMECs monolayer. The results are presented for a total of 582 nanoparticle‐modified T cells recorded in ten different movies and compared to 278 control T cells from five different movies for an observation time of 30 min.

To quantitatively describe the interaction of the nanoparticle‐decorated T cells with the pMBMEC monolayer under flow, each arrested T cell was assigned a behavioral category and the total number of T cells within a particular category was presented as a percentage of the total number of arrested T cells. T cells that entered or left the field of view during the recorded period were excluded from the analysis. T cells that continuously crawled were categorized as “crawling” and T cells that remained stationary while sending out protrusions as “probing.” The category “diapedesis” encompasses T cells that performed a complete diapedesis across the pMBMEC monolayer after crawling or probing. Finally, a category accounting for incomplete diapedesis after crawling or probing was defined as “incomplete diapedesis.” Figures S7–S10, Supporting Information, present sequences of images that illustrate each behavioral category. The corresponding movies are provided as Movies S7–S10, Supporting Information. A total of 582 nanoparticle‐modified T cells in ten different movies were compared to 278 control T cells from five different movies for an observation time of 25–30 min recorded at a rate of 6 frames per minute. **Figure** **9** shows the percentages of each behavioral category for unmodified and nanoparticle‐modified T cells. The results in Figure 9 indicate that the presence of a nanoparticle payload on the cell surface influences the behavior of the T cells. While after 30 min 65% of arrested unmodified T cells had crossed the TNF‐*α* stimulated monolayer, only 23% nanoparticle‐modified T cells managed to cross the pMBMEC monolayer during the same time. Concomitantly, the percentage of T cells that was identified as probing, crawling, or which underwent incomplete diapedesis significantly increased upon conjugation of nanoparticles to the T cell surface. In contrast to the results of the static transendothelial migration assay that were presented in Figure 7 and which revealed similar levels of extravasation of modified and non‐modified T cells, under flow conditions (Figure 9) ≈65% of the control T cells and ≈23% of the nanoparticle‐decorated T cells were found to undergo successful diapedesis. These apparent differences may partly be explained by the difference in the duration of the flow assay as compared to that of transendothelial assay under static conditions, which is typically performed for 6 h. In fact, this longer timeframe allowed nanoparticle‐decorated T cells to reach a similar extent of migration across the pMBMEC monolayer to that of unmodified control T cells. As nanoparticle‐decorated T cells did not show any impairment in arrest, probing, and crawling on the pMBMEC monolayer, the observed difference in T cell diapedesis across pMBMECs after 30 min under flow is most likely due to a delay caused by additional effort required from the T cells to squeeze their nanoparticle cargo through a transcellular pore or a paracellular gap across the pMBMEC monolayer.

The live cell imaging also helps to provide first hints to understand the differences in behavior between various nanoparticle‐decorated cells. Comparison of Figure 8 (and Movie_Figure S8, Supporting Information) and Figure S9, Supporting Information (Movie_Figure S9, Supporting Information) reveals marked differences in the size of the nanoparticle cluster at the uropod of the cell. Judging from the size of the nanoparticle cluster at the uropod of the cell that is shown in Figure S9, Supporting Information, as compared to that in Figure 8, the number of nanoparticles on the surface of the cell in the former image is larger as compared to the latter. This may suggest that diapedesis of the T cell in Figure S9, Supporting Information, is hindered by a very high surface concentration of nanoparticles on that particular T cell.

The time‐lapse live cell imaging experiments also allowed observing loss of nanoparticle cargo during diapedesis under flow conditions in several instances. For example, at least two different individual T cells were found to drop their nanoparticle load below the pMBMEC monolayer after completing diapedesis (Figure S11 and Movie_Figure S11, Supporting Information). Alternatively, the nanoparticle functionalized uropod was detached from the rest of the T cell during diapedesis leaving nanoparticles at the luminal surface of the pMBMEC monolayer (Figure S12 and Movie_Figure S12, Supporting Information). These observations confirm those made under static conditions namely that part of the nanoparticle payload may not be completely transported together with the T cells across the BBB.

### 2.4 In Vivo Analysis of Nanoparticle‐Decorated T Cells

**Figure 10:**
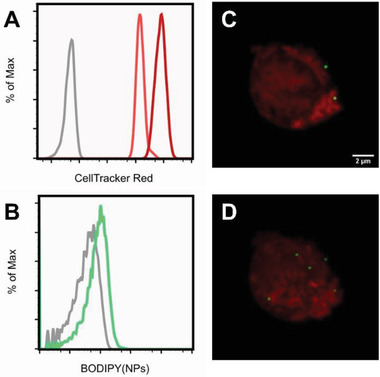
Flow cytometric analysis and confocal images of SJL‐PLP7 cells used for in vivo experiments. A) CellTracker Red associated channel of unmodified cells on day 3 (grey), cells labeled with CellTracker Red (2.5 µm) on day 3 (red) and cells labeled with CellTracker Red (2.5 µm) on day 4 (pale red). B) Nanoparticle‐associated channel of CellTracker Red labeled cells on day 4 (grey) and CellTracker Red labeled, nanoparticle‐modified cells on day 4 (green). C) Representative confocal microscopy section and D) representative 3D reconstruction of SJL‐PLP7 cells labeled with 2.5 µmCellTracker Red (red) and conjugated to nanoparticles (green) directly before in vivo injection. (*n* = 5, 4.2 ± 2.3 nanoparticles/cell).

Finally, the ability of the CD4^+^ T_EM_ cells to transport nanoparticles to the CNS was assessed in an in vivo model. To simultaneously monitor uptake of the T cells and the nanoparticles, these experiments were carried out with cells that were stained with CellTracker Red to visualize the cell body. As a consequence, the T cells used for the in vivo experiments were subjected to two consecutive chemical modification steps (CellTracker Red staining and nanoparticle surface conjugation), in contrast to the cells that have been used in all other experiments discussed so far, which were only exposed to a single, chemical surface modification reaction. Flow cytometry was used to monitor this two‐step chemical modification process and the average number of nanoparticles per cell was evaluated using confocal microscopy (**Figure** **10** and Figure S13, Supporting Information). Figure 10 and Figure S13A–C, Supporting Information, present the results that were obtained when T cells were first stained with 2.5 µm CellTracker Red and then modified with the maleimide‐functionalized nanoparticles at a ratio of 5000 particles/cell. In a second experiment (Figure S13D–F, Supporting Information), cells were stained with 1 µm CellTracker Red and then surface‐modified with the nanoparticles. Analysis of the confocal images indicated that the former reaction conditions result in 4.2 ± 2.3 nanoparticles/cell while the latter generates T cells that carry 12.0 ± 3.7 nanoparticles/cell. These nanoparticle cell‐surface concentrations are lower as compared to those reported earlier in this manuscript when the same cell‐surface conjugation reaction conditions were used. The reduced nanoparticle cell‐surface concentrations are due to the fact that CellTracker Red is a thiol reactive probe and thus competes with the nanoparticles that also target thiol groups. To facilitate the identification of diapedesed T cells in the brain sections with fluorescence microscopy (vide infra), T cells were stained with 2.5 µm CellTracker Red prior to nanoparticle surface conjugation.

**Figure 11:**
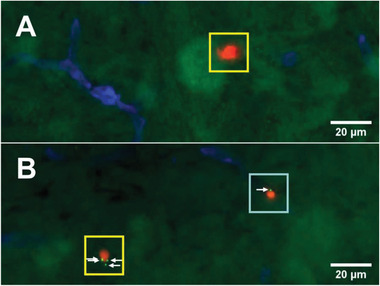
Widefield microscopy images of the brain parenchyma of mice after injection of A) CellTracker Red labeled SJL‐PLP7 cells and B) after injection of CellTracker Red labeled nanoparticle‐modified, SJL‐PLP7 cells. The endothelial and parenchymal basement membranes were stained for laminin (blue). Cells were labeled with 2.5 µmCTR (red) and conjugated to thiol reactive nanoparticles (green). T cells resident in the brain parenchyma are marked by a yellow box. T cells in the brain capillaries are marked by a light blue box. White arrows indicate the presence of nanoparticles on the cell surface.

The ability of the T cells to transport nanoparticles to the CNS in vivo was studied in TNF‐*α* pretreated C57BL/6 mice. In these experiments, 8.5 × 10^6^ CellTracker Red stained and nanoparticle‐loaded T cells in DPBS were systemically administered via a carotid artery catheter. As controls, mice were given 8.5 × 10^6^ CellTracker Red stained T cells as well as 1.05 × 10^6^ mPEG‐modified nanoparticles. After 5 h, mice were sacrificed and brain, liver, and spleen were collected. Migration of T cells and nanoparticles to the CNS was assessed by fluorescence microscopy analysis of brain sections. Per experiment, five brain sections with a thickness of 16 µm were analyzed. These sections were stained for laminin, which is a marker for the parenchymal basement membrane that separates the perivascular space from the CNS parenchyma, in order to distinguish circulating and CNS resident cells and nanoparticles. **Figure** **11** presents representative images of brain sections that were obtained from mice that were administered unmodified, CellTracker Red stained cells (Figure 11A) and nanoparticle‐modified, CellTracker Red stained cells (Figure 11B) (full size images of Figure 11A,B are included as Figures S14 and S15, Supporting Information). Analysis of these brain sections demonstrates T‐cell migration to the CNS both in case of administration of non‐surface‐modified, control T cells as well as when nanoparticle‐decorated T cells were injected. Cells that have migrated to the CNS are highlighted in Figure 11 with a yellow box. Highlighted in a light blue box in Figure 11B is a T cell that co‐localizes with the laminin stain, which indicates that this cell is found in the blood capillaries. In control experiments, where only nanoparticles were administered, the fluorescence micrographs did not provide any evidence for nanoparticle migration to the brain and CNS (Figure S16, Supporting Information). To quantify these analyses, for each experiment, the average number of T cells per section, the average number of CNS residing T cells per section, as well as the number of nanoparticle‐carrying T cells was determined. The results of these analyses are summarized in Table S1, Supporting Information. Analysis of the brain sections revealed the presence of 195 ± 48 T cells/section for mice that were administered nanoparticle‐decorated T cells. For mice that were administered non‐surface‐modified control T cells, 134 ± 25 cells/section were found. While the majority of the T cells was still found associated with the vasculature, 9.6 ± 6.1 (nanoparticle‐modified cells), resp. 4.0 ± 5.1 (non‐surface modified cells) had crossed the BBB and the glia limitans and resided in the CNS. When nanoparticle‐decorated cells were injected, 26% of the T cells that were found in the CNS still carried a nanoparticle payload. These cells carried 2.5 ± 2.3 nanoparticles per cell. Not surprisingly, fluorescent microscopy analysis of liver and spleen also revealed the presence of T cells and nanoparticles in these organs of the recipient mice (see Figures S17–S22, Supporting Information).

Unfortunately, a direct, quantitative comparison of the efficacy of T cell‐mediated nanoparticle delivery across the BBB in vivo versus in vitro described in the previous section is not possible due to the differences between the models and the different time scales of the experiments. Taken together, however, the transendothelial migration assays, the in vitro live cell imaging analysis, and the in vivo studies, unambiguously demonstrate that surface modification with the PEG‐functionalized polystyrene nanoparticles does not impair the ability of the CD4^+^ T_EM_ cells to cross the BBB in vitro or in vivo.

## 3 Conclusions

CD4^+^ T_EM_ cells possess unique abilities to extravasate across the BBB independent of their antigen specificity and both in the presence and in absence of neuroinflammation, which makes them potentially powerful carriers to facilitate and enhance transport of nanoparticles into the CNS. This study provides a first proof‐of‐concept that indicates the feasibility of CD4^+^ T_EM_ cells as carriers for cell‐mediated delivery to the CNS. The results presented here demonstrate that CD4^+^ T_EM_ cells can be surface‐modified using thiol–maleimide coupling chemistry with 200 nm diameter nanoparticles without imparting cell viability. Confocal microscopy experiments allowed to precisely and quantitatively characterize the localization and distribution of the nanoparticle payload on the cell surface and revealed that the nanoparticles are almost exclusively co‐localized with the cell membrane. In vitro experiments that were carried out both under static conditions as well as under physiological flow confirmed the ability of the nanoparticle‐decorated T cells to transport their cargo across a model BBB. While under flow after 30 min, 23% of the nanoparticle‐decorated and 65% of the non‐modified control cells were found to undergo successful diapedesis and the percentages of modified and control cells that had migrated across a monolayer of pMBMEC cells after a period of 6 h were identical. These results were confirmed in vivo, where nanoparticle‐decorated T cells were shown to enter the CNS parenchyma in comparable numbers to undecorated T cells delivering their nanoparticle cargo across the BBB.

## 4 Experimental Section

### Preparation of PEGylated Nanoparticles

In a typical procedure, 50 µL of 2% w/v solid 200 nm amine‐modified yellow–green polystyrene microspheres (≈0.85 µmol of —NH_2_ groups) were washed twice with MilliQ water (200 µL), resuspended in MilliQ water (50 µL) and sonicated for 10–15 min. Then, 5 eq (≈20 mg, 4.25 µmol) of maleimide‐PEG_5000_‐succinimidyl valerate (or methoxy‐PEG_5000_‐succinimidyl valerate for control unfunctionalized particles) were dissolved in MilliQ water (50 µL) and added to the particle solution. The reaction mixture (solid concentration, 1% w/v) was left on an orbital shaker (600 rpm) at room temperature for 60 min. Excess PEG was removed by three centrifugal washing cycles (30 000 x *g*) and particles were resuspended to the desired working concentration in MilliQ water and stored at 4 °C until used. Particle sizes and zeta potentials were measured in MilliQ water and in 1 mm sodium chloride solution (conductivity = 0.12 mS cm^−1^, pH = 6.5), respectively. Diameter of the methoxy‐PEGylated and maleimide‐functionalized PEGylated particles were 242 ± 3 nm (PDI: 0.036 ± 0.009) and 255 ± 4 nm (PDI: 0.106 ± 0.020), respectively. PEGylation resulted in a change of the zeta potential from +17.7 ± 1.3 mV for the amine‐modified nanoparticles to near neutral values for the PEGylated nanoparticles. The grafting density of PEG was estimated by ^1^H‐NMR analysis of the freeze‐dried particles dissolved in CDCl_3_ knowing the specific surface area of the FluoSpheres and was 0.2 chains nm^−2^. ^1^H‐NMR spectra of the methoxy‐PEGylated and the maleimide PEGylated nanoparticles as well as particle size distributions and zeta potentials are provided in Figures S1–S3, Supporting Information.

### T Cell‐Surface Modification with Maleimide‐Functionalized PEGylated Nanoparticles

T Cells were washed twice with DPBS (centrifuged at 250 x *g* for 7 min) and resuspended to a concentration of 30 × 10^6^ cells mL^−^ in DPBS. Then, 100 µL cell suspension (i.e., 3 × 10^6^ cells) was added to each well (Nunc, 96 well round bottom plate, low cell binding, Sigma‐Aldrich) containing either MilliQ water (100 µL), control particles (unfunctionalized methoxy‐PEGylated particles), or maleimide‐functionalized particles dispersed in MilliQ water at a concentration of 5000 particles/cell. The conjugation was performed at 37 °C for 30 min with gentle pipette mixing every 10 min. Unreacted maleimide groups were quenched by the addition of 20 µL of a 1 mm solution of *N*‐acetylcysteine and the cell suspension was left for another 10 min at 37 °C. Subsequently, cells were washed trice with ≈10 mL DPBS (centrifuged at 250 RCF for 7 min) to remove unbound and loosely bound particles.

## Conflict of Interest

The authors declare no conflict of interest.

## Author Contributions

M.A., B.E., and H.‐A.K. developed the concept and designed experiments. M.A., I.G., C.B., O.B., and R.G. conducted the experiments. O.B., R.G., and A.S. developed the confocal microscopy image analysis protocols. M.S., E.K., and G.E. designed and conducted the in vivo experiments. M.A., I.G., O.B., R.G., A.S., B.E., and H.‐A.K. analyzed the data. M.A, M.S., B.E., and H.‐A.K wrote the manuscript. All the authors provided critical comments on the manuscript.

## Supporting information

Supporting Information

Supplemental Movie 1

Supplemental Movie 2

Supplemental Movie 3

Supplemental Movie 4

Supplemental Movie 5

Supplemental Movie 6

Supplemental Movie 7

Supplemental Movie 8

Supplemental Movie 9

Supplemental Movie 10

Supplemental Movie 11
