# Supplementary material for: T Cell‐Mediated Transport of Polymer Nanoparticles across the Blood–Brain Barrier
Source: Adv Healthc Mater. 2020 Nov 25;10(2):2001375. doi: 10.1002/adhm.202001375 (PMC11469241; doi:10.1002/adhm.202001375)
Supplement: Supplementary file 1 — Supporting Information [file ADHM-10-2001375-s011.pdf]

© 2020 Wiley-VCH GmbH

ADVANCED
HEALTHCARE
MATERIALS

Supporting Information

for *Adv. Healthcare Mater.*, DOI: 10.1002/adhm.202001375

T Cell-Mediated Transport of Polymer Nanoparticles across the Blood-Brain Barrier

Maxime Ayer, Markus Schuster, Isabelle Gruber, Claudia Blatti, Elisa Kaba, Gaby Enzmann, Olivier Burri, Romain Guiet, Arne Seitz, Britta Engelhardt, and Harm-Anton Klok**

SUPPORTING INFORMATION:

T Cell-Mediated Transport of Polymer Nanoparticles across the Blood-Brain Barrier

Maxime Ayer,[1] Markus Schuster,[1] Isabelle Gruber,[2] Claudia Blatti,[2] Elisa Kaba,[2] Gaby Enzmann,[2] Olivier Burri,[3] Romain Guiet,[3] Arne Seitz,[3] Britta Engelhardt,[2,] Harm-Anton Klok[1,*]*

[1]École Polytechnique Fédérale de Lausanne (EPFL), Institut des Matériaux and Institut des Sciences et Ingénierie Chimiques, Laboratoire des Polymères, Bâtiment MXD, Station 12, CH-1015 Lausanne, Switzerland.

[2]Theodor Kocher Institute, University of Bern, Freiestrasse 1, CH-3012 Bern, Switzerland.

3 École Polytechnique Fédérale de Lausanne (EPFL), Faculté des sciences de la vie, Bioimaging and optics platform, Bâtiment AI, Station 15, CH-1015 Lausanne, Switzerland.

*To whom correspondence should be addressed: bengel@tki.unibe.ch ; harm-anton.klok@epfl.ch.

EXPERIMENTAL SECTION

MATERIALS

Amine-modified yellow-green FluoSpheres (d = 200 nm), CellTrace Violet, CellTracker Red, WGA-Texas Red conjugate, WGA-Alexa Fluor647 conjugate, Annexin V-Alexa Fluor 647 conjugate, Annexin buffer and Prolong Gold mounting media, Dulbecco's phosphate-buffered saline (DPBS), RPMI 1640 and FBS were purchased from Thermofischer Scientific. Methoxy-PEG_{5000}-succinimidyl valerate and maleimide-PEG_{5000}-succinimidyl valerate were obtained from Laysan Bio Inc.. Poly(L-lysine) (0.1 % w/v solution), 4′,6-diamidine-2′-phenylindole dihydrochloride (DAPI) and 1 mM Staurosporine solution in DMSO were purchased from Sigma-Aldrich. Rabbit anti-pan Laminin (Z0097) was purchased from DAKO. Goat anti-rabbit AMCA (111-156-045) was purchased from Jackson Immuno Research.

METHODS

Particle sizes and zeta potentials were measured by dynamic light scattering (DLS) using a Zetasizer Nano Zs instrument (Malvern). ^{1}H-NMR spectra were recorded on Bruker Avance III 400 MHz spectrometer. Flow cytometry analysis was performed on a Beckmann Coulter Gallios instrument. Confocal microscopy images were recorded on a Zeiss LSM700 Inverted microscope (Carl Zeiss, Feldbach, Switzerland). Fluorescence microscopy images were recorded on a Nikon Eclipse E600 microscope connected to a Nikon Digital Camera DXM1200F with the Nikon NIS-Elements software (Nikon, Egg, Switzerland). Cell-counting for binding assay was performed using an Olympus CKX41 inverted microscope equipped with a 10 mm x 10 mm / 10 divisions counting reticle and a 20x objective.

PROCEDURES

Cell lines and cell cultures

$CD4^+$ effector/memory proteolipid protein (PLP) peptide aa139-153 specific T cells (line SJL/PLP7) were cultured as previously described.[1] In brief, SJL/PLP7 T cells were cultured in RPMI 1640 glutamax medium (Gibco) supplemented with 10% fetal bovine serum (FBS) (Gibco), 1%

penicillin/streptomycin (Gibco), 1% non-essential amino acid (Gibco), 1% Na-pyruvate (Gibco), 0.4% β-mercaptoethanol (Gibco) and 1% IL-2 supernatant (self-made). Cells were typically used for modification and in functional assays at days 3 or 4 after *in vitro* antigen-specific restimulation. If transferred into naïve syngeneic recipients SJL/PLP7 transfers experimental autoimmune encephalomyelitis (EAE). The molecular mechanisms employed by SJL/PLP7 cells to cross the BBB have been characterized in detail *in vivo* and *in vitro*.[1,2,3]

Isolation and culture of *primary mouse brain microvascular endothelial cells (pMBMECs)* was performed as described before.[4,5,6] In brief, cortices from 6-8 weeks old C57BL/6 mice were isolated by removing the cerebellum, optic nerves and brain white matter. Meninges were then removed using dry cotton swabs. Preparations were ground using a Dounce homogenizer with a large clearance pestle of 0.06 – 0.08 mm in wash buffer (HBSS containing 10 mM HEPES and 0.1% BSA). The resulting homogenate was mixed with 30% dextran (v/v, molecular weight 100 000 – 200 000 Da) in wash buffer. This suspension was centrifuged at 3000 g for 25 min at 10 °C. The neural component and the dextran layer were discarded, the pellet containing the vascular component was filtered through a nylon mesh with 60 µm pore size. The capillary-enriched filtrate was digested in collagenase/dispase (2 mg/mL) in wash buffer supplemented with DNase I (10 µg/mL) and TLCK (Tosyl-L-lysyl-chloromethane hydrochloride) (0.147 µg/mL) for 30 min at 37 °C. Digestion was stopped by adding an excess of wash buffer and the suspension filtered through a nylon mesh of 20 µm pore size. The resulting digested capillary suspension was seeded onto a tissue culture-treated dish or insert. Culture medium was DMEM supplemented with 20% FBS, 2% sodium pyruvate, 2% non-essential amino acids, gentamycin (50 µg/mL), basic fibroblast growth factor (1 ng/mL) and for the first 48 h with puromycin (4 µg/mL). 24 h after plating, red blood cells, cell debris and non-adherent cells were removed by washing with medium. Afterwards, the medium was changed every second day and pMBMECs were used on day 7 or 8 after isolation.

Proliferation assay

T-cell proliferation was monitored with CellTrace Violet according to the manufacturer's protocol. In short, cells were washed once and resuspended into DPBS at a concentration of 1 x 10^6 cells/mL. 1 µL of CellTrace Violet stock solution in DMSO (5 mM) was added per milliliter of cell suspension and left for 20 minutes at 37 °C in the dark. Excess dye was removed by addition of approx. 5 times the staining volume of complete growth medium. Cells were then pelleted, washed once with DPBS and resuspended in complete growth medium until use for cell-surface modification experiments. Fluorescence intensities were measured by flow cytometry, directly after surface modification and 24 hours after modification.

Confocal microscopy and image analysis

Nanoparticle modified and CellTrace Violet stained T cells were additionally stained with a membrane marker, WGA-Texas Red X/ WGA-Alexa Fluor647 for 30 minutes on ice in DPBS at a concentration of 1 x 10^6 cells/mL. Cells were then seeded on a poly(L-lysine) coated borosilicate glass precision microscopy coverslip (12 mm diameter, 0.17 mm thickness; Carl Roth GmbH), washed twice with DPBS and fixed for 10 minutes with a 4% paraformaldehyde solution in DPBS at room temperature. Fixed cells were finally mounted on a microscopy glass slide with Prolong Gold. The slides were left to cure overnight before images were acquired on a Zeiss LSM700 microscope with a 63x/1.4NA lens. Voxel sizes were optimized for deconvolution (XYZ 30 nm x 30 nm x 130 nm). Digital gain and pinhole size were set as follows for each channel 1.0 and 49 µm for the whole cell channel (CellTrace Violet); 1.25 and 48 µm for the membrane channel (WGA-Texas Red-X); 1.3 and 47 µm for the nanoparticle channel (BODIPY). Gain was optimized for each z-stack.

Cell viability assay

Cell viabilities were assessed using Annexin V – Alexa Fluor 647 and DAPI as apoptotic and necrotic markers, respectively. Briefly, unmodified or surface-modified T cells were washed once with DPBS and 0.3 x 10^6 cells were resuspended in Annexin buffer containing DAPI (1 µg/mL) at a concentration of 1 x 10^6 cells/mL. 15 µL Annexin V - Alexa Fluor 647 conjugate were added to the

cell suspension and cells were incubated at room temperature for 15 minutes in the dark. Subsequently, 400 µL Annexin buffer was added and cells were analyzed by flow cytometry directly after modification. The assay was repeated 12 hours and 24 hours after modification. As a positive control for apoptosis, T cells were incubated in complete growth medium supplemented with staurosporine (1 µM) for at least 6 hours. 2D scatter plots of the flow cytometry analysis, which represent the forward vs side scatter and Annexin V – Alexa Fluor 647 vs DAPI associated fluorescence are illustrated in **Figure S5**. The results for the untreated T cells and the staurosporine treated T cells were used to gate the cell populations. Quadrants Q2, Q3 and Q4 in the 2D scattered plots of Annexin V - Alexa Fluor 647 vs DAPI associated fluorescence are gated as apoptotic/necrotic, apoptotic and viable, respectively.

Binding assay

ICAM-1 coated slides were prepared as previously reported.[7] In brief, standard 12 well diagnostic slides (ER-202W-CE24, ThermoFisher Scientific) were coated with a protein A (BioVision, Lausen, Switzerland) solution (20 µg/mL) in PBS (pH 9) for 1 h at 37 °C. The protein A incubation was followed by three PBS washes and subsequently a blocking step using 1.5% bovine serum albumin (BSA) in PBS overnight at 4 °C. Wells were then washed once with PBS (pH 7.4) and protein A was exposed to recombinant purified cell-adhesion molecule (100 nM) mouse ICAM-1-Fc chimera (R&D Systems, Abingdon, U.K.) for 2 hours at 37 °C and finally the wells were blocked with 1.5% BSA in PBS for 30 min at room temperature and washed once with PBS before used in a binding assay. As a control DNER-Fc (R&D Systems, Abingdon, U.K.) chimera was used instead of mouse ICAM-1-Fc chimera. For the binding assay, T cells were collected at 10 x 10^6 cells/mL in migration assay medium (MAM: DMEM, 25 mM HEPES, 5% FBS, 2% L-glutamine) and 1 x 10^5 cells were added to each well and the slide was incubated for 30 minutes at room temperature on a rotating platform. The slides were finally washed twice by dipping them into PBS and fixed for 2 h in 2.5% v/v glutaraldehyde in PBS. The number of adherent cells was evaluated by counting the number of bound cells per field of view using a 20x objective mounted on an Olympus CKX41 inverted

microscope equipped with a 10 mm x 10 mm / 10 divisions counting reticle. Each dot in **Figure 6** **r**epresents a single cell count from the diagonal of the reticle. To determine the number of adherent T cells, 3 fields per well (i.e. per replicate) were evaluated in a total of two independent experiments performed in triplicate).

Transendothelial migration assay under static conditions

Transmigration assays were performed as described before[8] using a two chamber Transwell system. In brief, pMBMECs were seeded on 6.5 mm filter inserts with a 5 µm pore size (Costar, Bodenheim, Germany) previously coated with laminin and matrigel. In order to prevent the pMBMECs from sprouting through the pores of the filter, they were grown to confluency without medium in the lower compartment. For experiments with stimulated monolayers, prior to the experiment, pMBMECs were stimulated with recombinant mouse tumor necrosis factor alpha, TNF-α (10 ng/mL) for 16 h. At the beginning of the transmigration assay, pMBMEC inserts were washed twice with migration assay medium (MAM: DMEM (Gibco), 2 % L-Glutamine, 25 mM HEPES (Gibco), 5 % FBS (Gibco)) before being transferred into a new 24-well Costar plate well containing MAM (600 µL). Then, MAM (100 µL) containing 1.0 x 10^5 T cells were added per insert and T cells were allowed to transmigrate for 6 h at 37 °C. Additionally, aliquots of 1.0 x 10^5 T cells were kept in MAM (600 µL) and used as representative for the input. The number of transmigrated T cells and the number of T cells in the input samples were assessed by flow cytometry (FACS calibur) using BD Trucount tubes (BD biosciences). The percentage of migrated T cells was calculated referring to the inputs as 100 %. Finally, the inserts were washed twice in PBS and fixed in 1% PFA. Fixed inserts were stained with phalloidin-rhodamin and DAPI and mounted on glass slides in order to confirm the confluency of the endothelial monolayer of each filter after the assay.

Multistep T-cell extravasation under physiological flow

In vitro live cell imaging of T cell extravasation across pMBMECs cultured on matrigel coated cell culture surfaces (µ-dish 35 mm-low, ibidi Vitaris, Baar, Switzerland) was performed as described

before.[9] The pMBMECs were stimulated with recombinant mouse tumor necrosis factor alpha (TNF-α, 10 ng/mL) for 16-24 h prior to the experiment. A small custom-made flow chamber was used here.[9] The inlet tubing of the flow chamber was filled with MAM and the flow chamber placed on the endothelial cells. Flow was then applied by connecting the outlet tubing to a syringe automatically drawn up by a precision pump (Harvard Apparatus, Holliston, MA, USA). For microscopic imaging, the assembled flow chamber was placed on the stage of an inverted microscope (AxioObserver.Z1, Carl Zeiss, Feldbach, Switzerland) equipped with a temperature-controlled chamber (37 °C). Image acquisition was performed through computer control using the ZEN software (Carl Zeiss) at a rate of 10 images per min and with a 20-fold (Objective LD "Plan-Neofluar" 20×/0,4 Korr Ph2 M27) magnification using a monochrome CCD camera (AxioCam MRmRev, Carl Zeiss). Aspiration of T cells from a reservoir via the inlet tubing was performed at a flow rate corresponding to a wall shear stress of 1.5 dyne/cm^2 until the T cells appear in the field of view. To allow settling of the T cells on the endothelial surface, the flow rate was reduced to a level that corresponds to a wall shear stress of 0.2 dyne/cm^2 and accumulation was terminated after 5 min by increasing the flow to generate a wall shear stress of 1.5 dyne/cm^2 (mimicking physiological flow conditions). Image recording in time-lapse mode was started at the beginning of the accumulation phase and continued for up to 30 min. The dynamic interaction of T cells with the endothelium was evaluated by assigning a migratory phenotype to each T cell using ImageJ software (National Institute of Health, Bethesda, MD, USA). To this end, each arrested T cell is assigned a digit 30 sec after the end of the accumulation phase. The behavior of each individual T cell was analyzed throughout the complete movie and then accordingly assigned to one category. T cells that continuously crawled were categorized as "Crawling". T cells that were probing without crawling or diapedesis are categorized as "Probing". T cells that crossed the BBB after having crawled to or having probed at a site of diapedesis were categorized as "Diapedesis". T cells that had partially crossed the endothelial monolayer were categorized as "Incomplete diapedesis". Arrested T cells that enter or left the field of view during the recording time were excluded from the evaluation.

T cell labeling with CellTracker Red

To track them in frozen tissue sections, T cells were labeled with CellTracker Red according to the manufacturer's protocol 1 day before nanoparticle conjugation and injection into mice. In short, cells were washed twice with DPBS and were resuspended in a solution of CellTracker Red in DPBS (1-2.5 μM) at a cell concentration of 1 x 10^6 cells/mL. After incubation for 30 minutes at 37 °C in the dark, cells were washed twice in TCGF, resuspended in TCGF at a concentration of 0.3 x 10^6 cells/mL and re-plated until further use. Fluorescence intensities were measured by flow cytometry directly after CellTracker Red labeling and directly after conjugation of the CellTracker Red labeled cells to nanoparticles.

Injection of nanoparticle modified T cells into the carotid artery of mice

Mice were housed in individually ventilated cages under specific pathogen-free conditions at 22 °C with free access to chow and water. Animal procedures executed were approved by the Veterinary office of the Canton Bern (permission number 31/17). T cells were labeled with CellTracker Red 1 day before injection and nanoparticle-modified directly before injection using the protocols described above. 100 μL CellTracker Red labeled T cells (8.5 x 10^6), nanoparticle functionalized CellTracker Red labeled T cells (8.5 x 10^6) and free nanoparticles in DPBS (1.05 x 10^9) were injected into the carotid artery of C57/Bl6 mice (female, 8 weeks old, 1 animal per condition) using the following protocol. Mice were injected with TNF-α (1 μg, ip) at least 4 h before surgery and with Buprenorphine (7.5 μg, ip) at least 30 min before surgery. Directly before surgery, mice were anaesthetized in a flow chamber using isoflurane (4.5%, 600 mL/min). Anesthesia was maintained during the surgery using isoflurane (3%, 220 mL/min). The eyes were protected with Lacrinorm® and 0.9% NaCl (1 mL, ip) was injected to prevent dehydration of the mice. The carotid artery was assessed via a 0.5 cm incision at the trachea and isolated using 3 sutures. A catheter was inserted in a small incision at the carotid artery, the blood flow was opened by loosening the heart side wire and the knot on the cranial side and samples were slowly applied using the catheter. The catheter was flushed with 0.9% NaCl (50 μL), removed together with all wires and the skin was closed with 2-3

sutures. Mice were allowed to recover from anesthesia, sacrificed after 3-5h and perfused with PFA (1%, 10 mL). Brain, liver and spleen were collected, embedded in OCT resin and frozen at -80 °C.

Immunofluorescence staining of tissue sections

For fluorescence imaging, tissue sections (16-20 µm) were cut on a cryostat at -20 °C, mounted on SuperFrost Plus® microscopy slides (Thermo Scientific) and dried overnight in the dark. The area around the dried sections was restricted using a DAKO Pen®. Sections were post fixed using PFA (2%, 150 µL) for 10 min at room temperature. The PFA was discarded and sections were washed 3x for 5 min with TBS (50 mM Trizma Base, 150 mM NaCl, 1 mM $CaCl_2$ x $2H_2O$, pH = 7.4). After incubation of the sections with blocking buffer (150 µL) (TBS, 5% w/V skimmed milk, 0.3% V/V Triton X-100, 0.04% w/V NaN_3) for 20 min at room temperature, a primary antibody (rabbit anti-pan Laminin, DAKO Z0097 or rabbit IgG, R&D Systems, AB-105-C; 5 µg/mL) in blocking buffer (150 µL) was applied for 1 h at room temperature. Unbound primary antibodies were removed by 3 washes with TBS for 5 min and a secondary antibody (goat anti rabbit AMCA, Jackson, 111-156-045; 10 µg/mL) in blocking buffer (150 µL) was applied for 1 h at room temperature. Unbound secondary antibodies were removed by 3 washes with TBS for 5 min and sections were mounted on MOWIOL® and dried overnight before imaging.

Statistical Analysis

Significant differences between two samples were determined using the Student's t-test using the software "Origin" (Origin Pro 2016, OriginLab Corp., Northampton, MA, USA). Statistical differences between two groups were considered for *p*-values < 0.05 and marked by an asterix. Results from in-vitro experiments are reported based on two separate experiments performed in triplicate.

REFERENCES

[1] B. Engelhardt, M. Laschinger, M. Schulz, U. Samulowitz, D. Vestweber, G. Hoch, *J. Clin. Invest.* **1998**, *102*, 2096.

[2] M. Abadier, N. Haghayegh Jahromi, L. Cardoso Alves, R. Boscacci, D. Vestweber, S. Barnum, U. Deutsch, B. Engelhardt, R. Lyck, *Eur. J. Immunol.* **2015**, *45*, 1043.

[3] P. Vajkoczy, M. Laschinger, B. Engelhardt, *J. Clin. Invest.* **2001**, *108*, 557.

[4] C. Coisne, L. Dehouck, C. Faveeuw, Y. Delplace, F. Miller, C. Landry, C. Morissette, L. Fenart, R. Cecchelli, P. Tremblay, B. Dehouck, *Lab. Invest.* **2005**, *85*, 734.

[5] R. Lyck, N. Ruderisch, A. G. Moll, O. Steiner, C. D. Cohen, B. Engelhardt, V. Makrides, F. Verrey, *J. Cereb. Blood Flow Metab.* **2009**, *29*, 1491.

[6] O. Steiner, C. Coisne, B. Engelhardt, R. Lyck, *J. Cereb. Blood Flow Metab.* **2011**, *31*, 315.

[7] G. Martin-Blondel, B. Pignolet, S. Tietz, L. Yshii, C. Gebauer, T. Perinat, I. Van Weddingen, C. Blatti, B. Engelhardt, R. Liblau, *Eur. J. Immunol.* **2015**, *45*, 3302.

[8] R. K. Röhnelt, G. Hoch, Y. Reiss, B. Engelhardt, *Int. Immunol.* **1997**, *9*, 435.

[9] C. Coisne, R. Lyck, B. Engelhardt, *Fluids Barriers CNS* **2013**, *10*, 7.

Table S1. Quantitative analysis of brain sections (n = 5) of C57Bl6 mice after injection of non-modified and nanoparticle-modified, CellTracker Red labeled SJL-PLP7 T cells.

Condition	*Non-modified cells*	*Nanoparticle-decorated cells*
Average number of T cells per section	130 ± 25	195 ± 48
Average number of T cells located in the CNS per section	4.0 ± 5.1	9.6 ± 6.1
Average number of nanoparticles/cell on the surface of T cells that are located in the CNS	-	2.5 ± 2.3

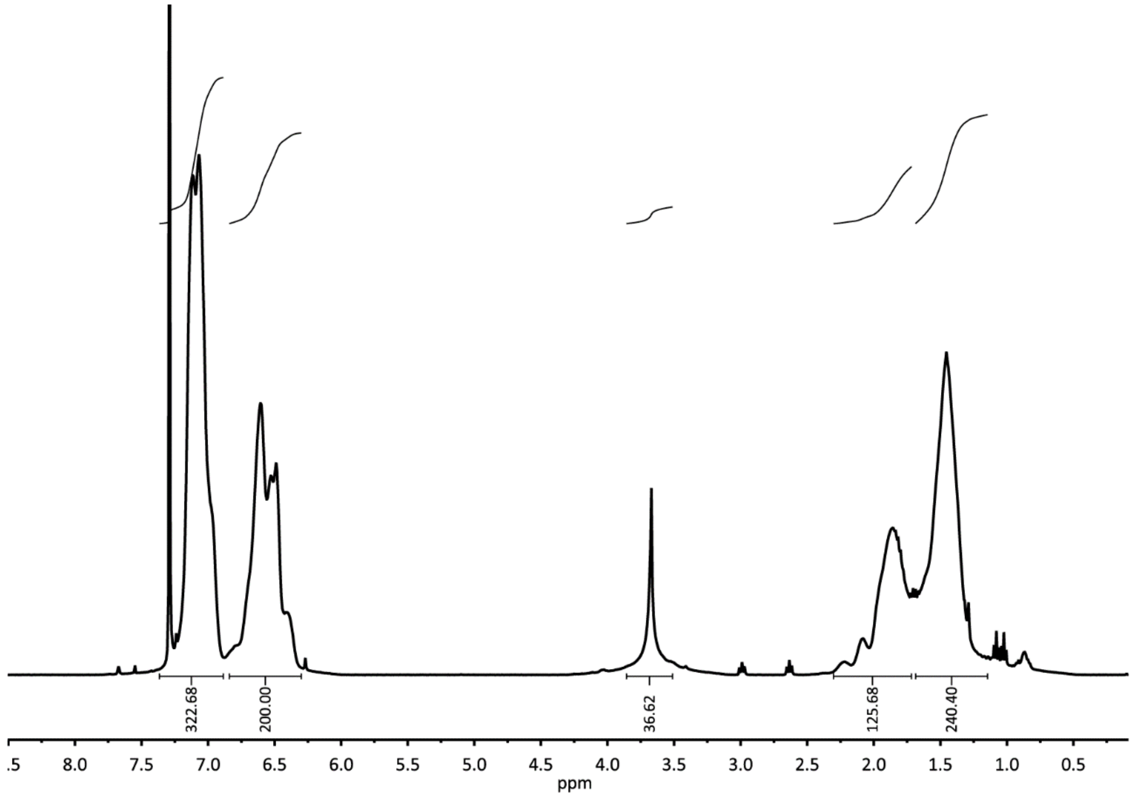

Figure S1. ¹H-NMR spectrum of freeze-dried methoxy-PEGylated amine-modified FluoSpheres dissolved in $CDCl_3$.

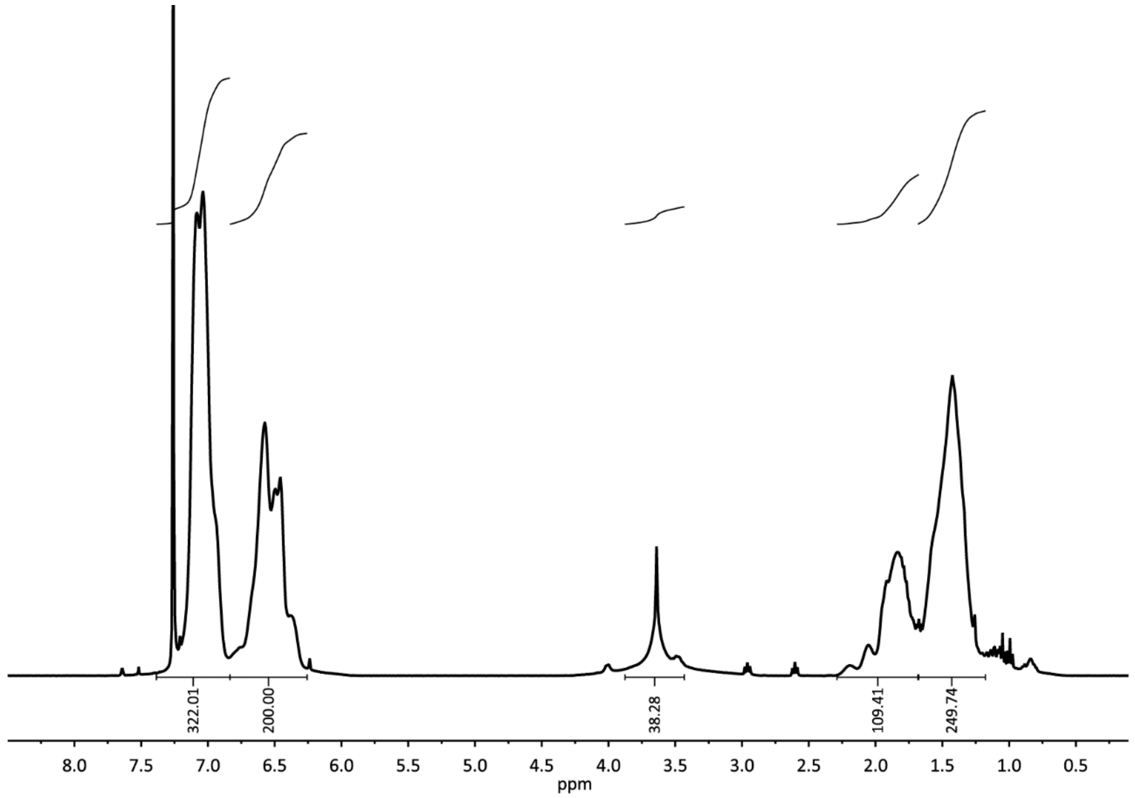

Figure S2. ¹H-NMR spectrum of freeze-dried maleimide functionalized PEGylated amine-modified FluoSpheres dissolved in $CDCl_3$.

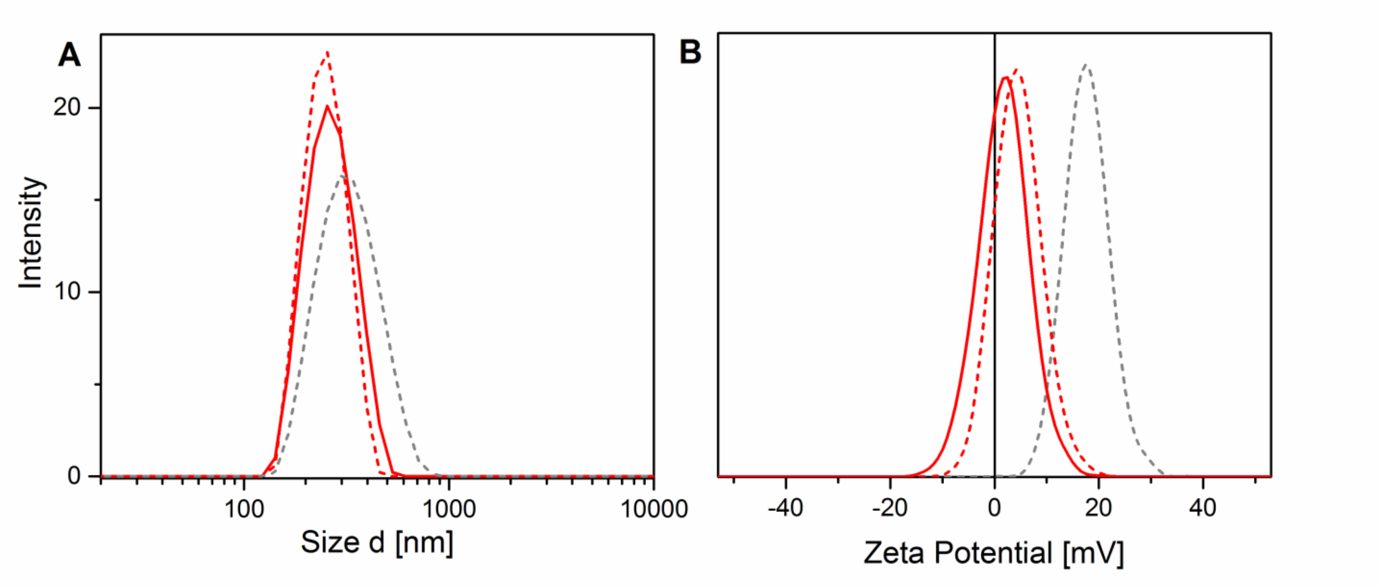

Figure S3. Particle size distribution **(A)** and zeta potential **(B)** of maleimide functionalized PEGylated polystyrene nanoparticles, (solid red), methoxy-PEGylated polystyrene nanoparticles (dash red) and amine-modified polystyrene nanoparticles (dash gray, commercial product).

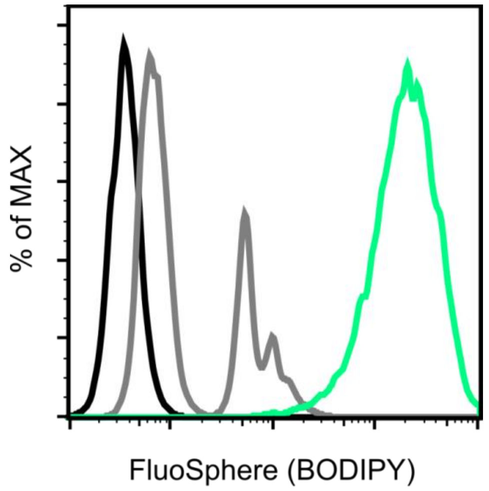

Figure S4. Flow cytometry results showing the specific binding of maleimide functionalized PEGylated nanoparticles to the T-cells. The figure shows histograms of scatter gated live T cells for nanoparticle associated fluorescence. Control untreated T cells are shown in black; T cells treated with methoxy-PEGylated nanoparticles in grey and T cells treated with maleimide-functionalized PEGylated nanoparticles in green. This result is representative of two independent experiments performed in triplicate.

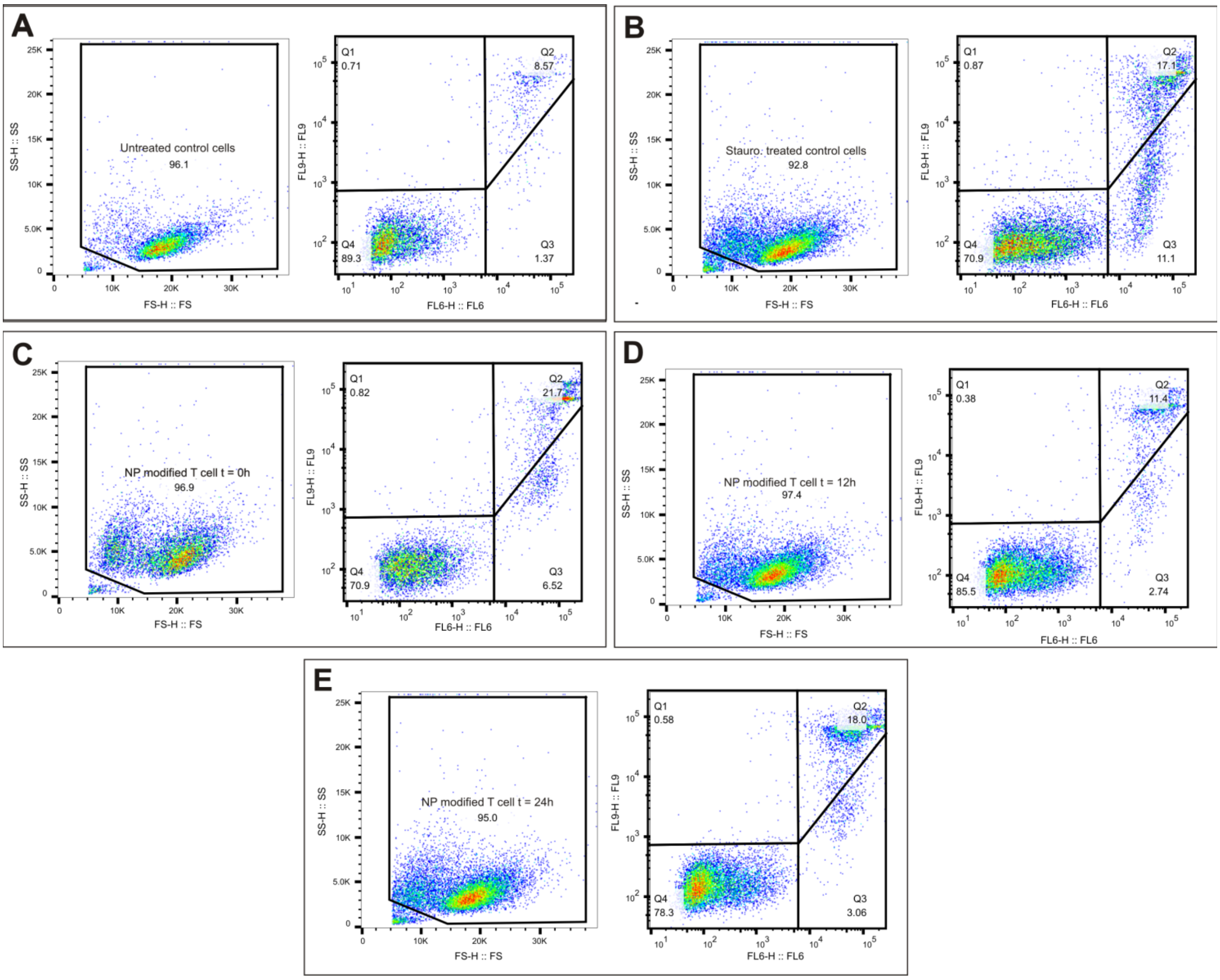

Figure S5. Annexin V-Alexa 647 / DAPI viability assay. For each box: (Left) Forward and side scatter gated population for viability assay and (right) plot of fluorescence of Annexin V-Alexa 647 (FL-6) versus fluorescence of DAPI (FL-9) (A) untreated control T cells. (B) Staurosporine treated (for 6 hours) control T cells. (C) Nanoparticle modified T cells directly after functionalization. (D) Nanoparticle modified T cells 12 hours after functionalization. (E) Nanoparticle modified T cells 24 hours after functionalization. Quadrant Q4 show live T cells, Q3 apoptotic T cells and Q2 apoptotic/necrotic T cells as gated from the two controls: untreated control T cells (A) and staurosporine treated T cells (B) plots.

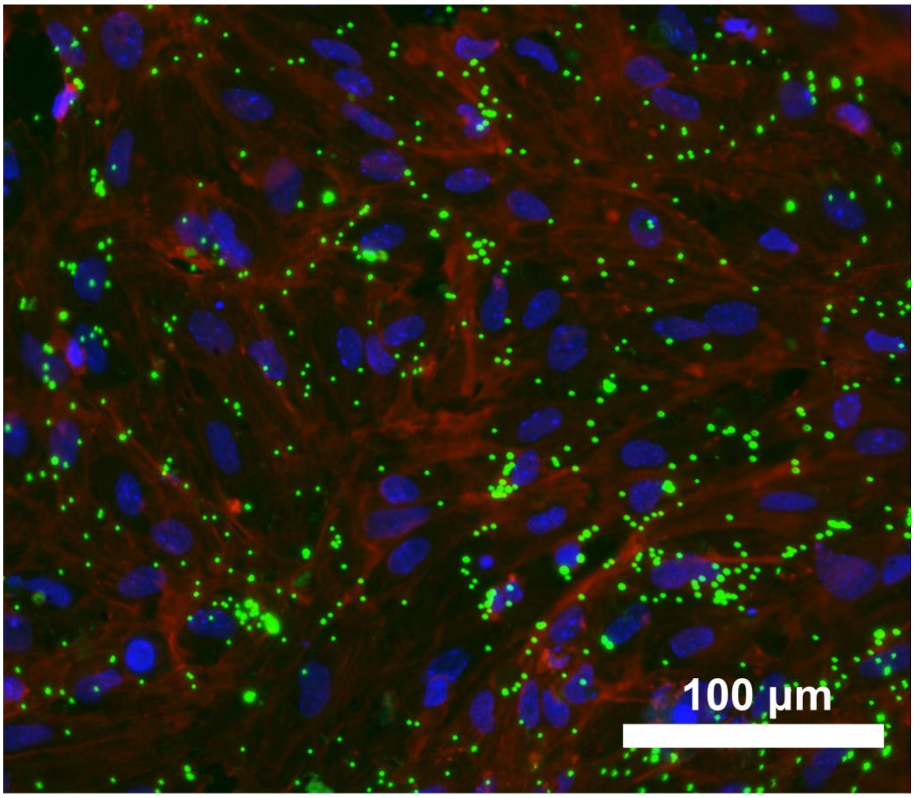

Figure S6. Fluorescence image of a pMBMEC monolayer stained with DAPI (blue) highlighting the nuclei and phalloidin-rhodamin (red) highlighting the actin skeleton after transmigration of nanoparticle (green) modified T cells under static conditions. Nanoparticles that were shed from the T cell surface during transmigration remained associated with the pMBMEC monolayer. Scale bar = 100 μm.

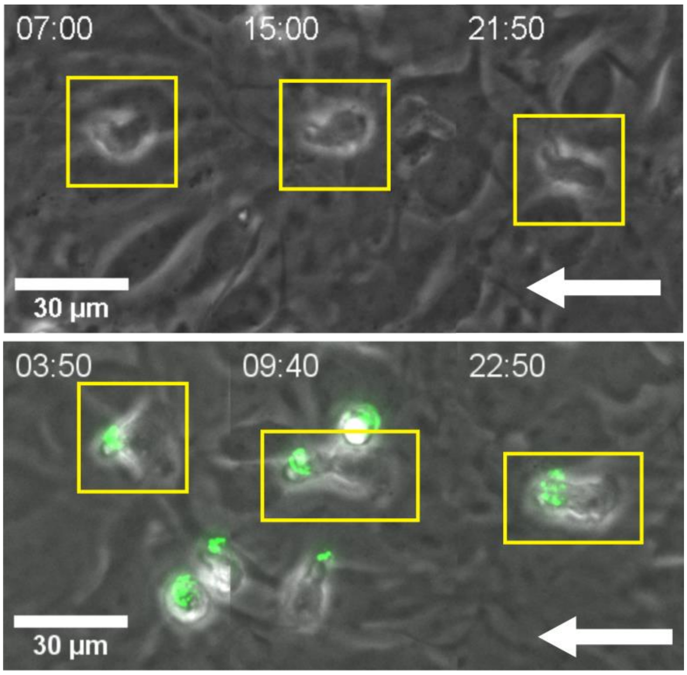

Figure S7. T cell crawling on pMBMECs under physiological flow. A sequence of three images recorded during time-lapse live cell imaging of (top) a control T cell and (bottom) a nanoparticle

(green) decorated T cell crawling on a pMBMEC monolayer under physiological flow is shown. The yellow rectangles indicate the position of the cell of interest as a function of time. The white arrow indicates the direction of the flow. Time is indicated in left corners of each images as [min:sec].

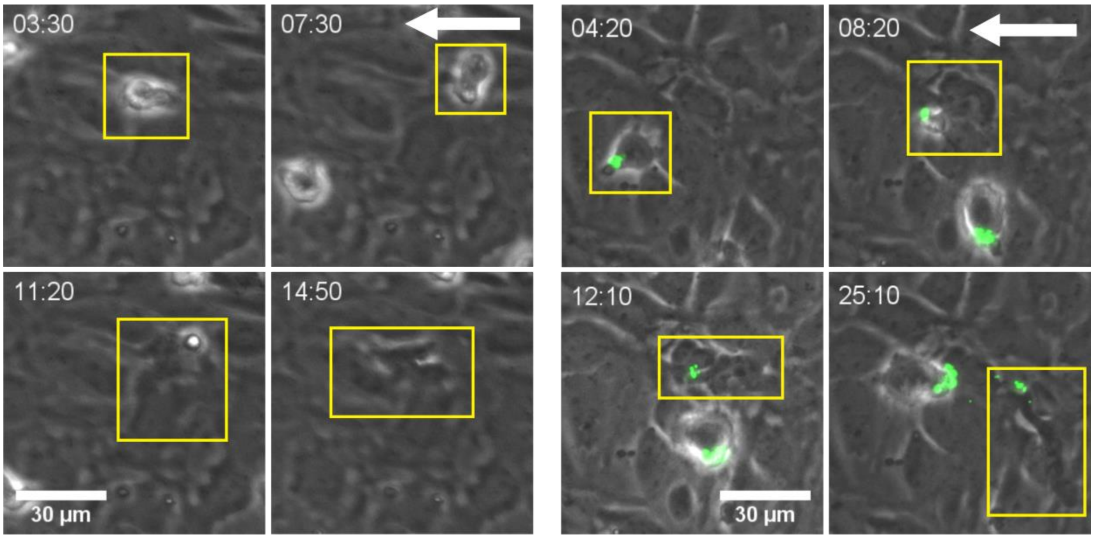

Figure S8. T-cell diapedesis. A sequence of four images recorded during time-lapse live cell imaging of a control T cell (left) and a nanoparticle modified T cell (right) performing complete diapedesis across a pMBMEC monolayer under physiological flow is shown. The yellow rectangles indicate the position of the cell of interest as a function of time. The white arrow (top right corner) indicates the direction of the flow. Time is indicated in left corners of each images as [min:sec].

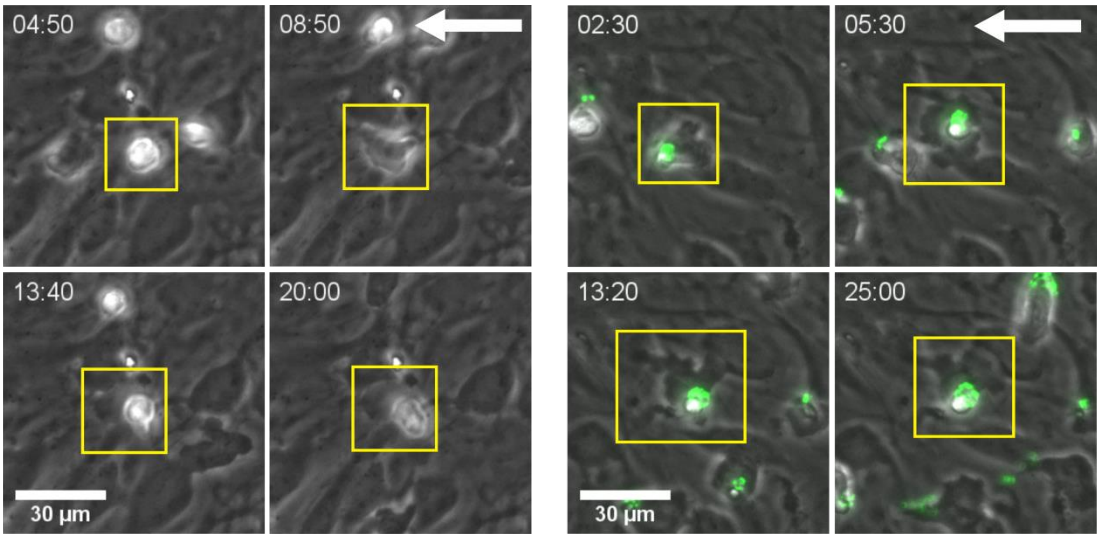

Figure S9. Incomplete diapedesis of T cells. A sequence of four images recorded during time-lapse live cell imaging of (left) a control T cell and (right) a nanoparticle (green) decorated T cell performing an incomplete diapedesis across a pMBMEC monolayer under physiological flow is shown. In both cases, the uropod remains on the luminal side of the pMBMEC monolayer. Nanoparticles are clustering at the uropod in the image sequence on the right. The yellow rectangles indicate the position of the cell of interest as a function of time in both left and right image series. The white arrow (top right corner) indicates the direction of the flow. Time is indicated in left corners of each images as [min:sec].

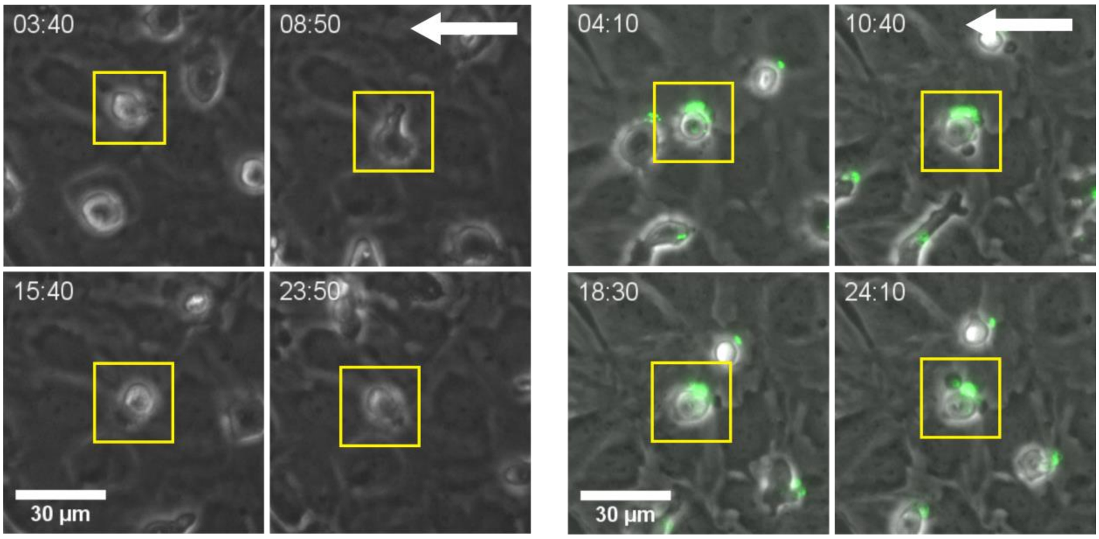

Figure S10. T cell probing. A sequence of four images recorded during time-lapse live cell imaging of (left) a control T cell and (right) a nanoparticle (green) decorated T cell probing on the luminal side of a pMBMEC monolayer under physiological flow is shown. The yellow rectangles indicate the position of the cell of interest as a function of time in both left and right image series. The white arrow (top right corner) indicates the direction of the flow. Time is indicated in left corners of each images as [min:sec].

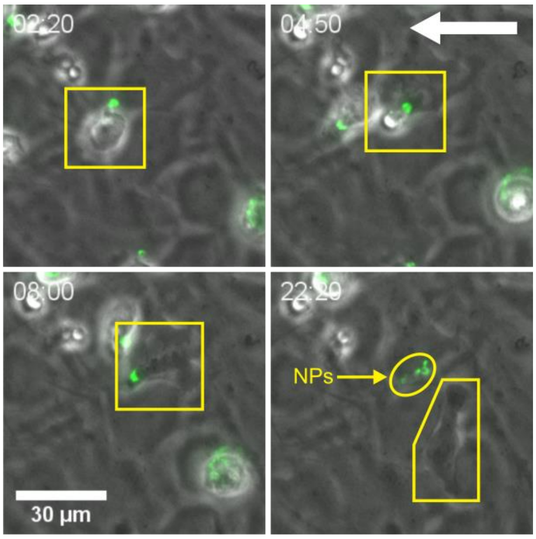

Figure S11. Nanoparticle dropped after diapedesis. A sequence of four images recorded during time-lapse live cell imaging of a nanoparticle (green) decorated T cell dropping its nanoparticle cargo after diapedesis across a pMBMEC monolayer under physiological flow is shown. The yellow rectangles indicate the position of the cell of interest as a function of time. At the end of the sequence (image at 22:20) dropped nanoparticles (NPs) are marked with a yellow circle and the T cell is marked with a yellow polygon. The white arrow (top right corner) indicates the direction of the flow. Time is indicated in left corners of each images as [min:sec].

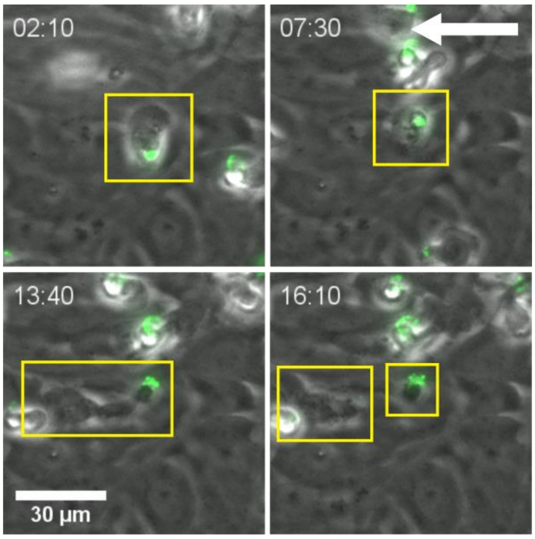

Figure S12. T cell breaking apart, losing its nanoparticle functionalized uropod. A sequence of four images recorded during time-lapse live cell imaging of a nanoparticle (green) decorated T cell losing its nanoparticle decorated uropod after diapedesis across a pMBMEC monolayer under physiological flow is shown. The yellow rectangles indicate the position of the cell of interest as a function of time in the image series. On the image taken at 16.10 the modified T cell is broken apart and the functionalized uropod is left on the luminal side of the pMBMEC monolayer The white arrow (top right corner) indicates the direction of the flow. Time is indicated in left corners of each images as [min:sec].

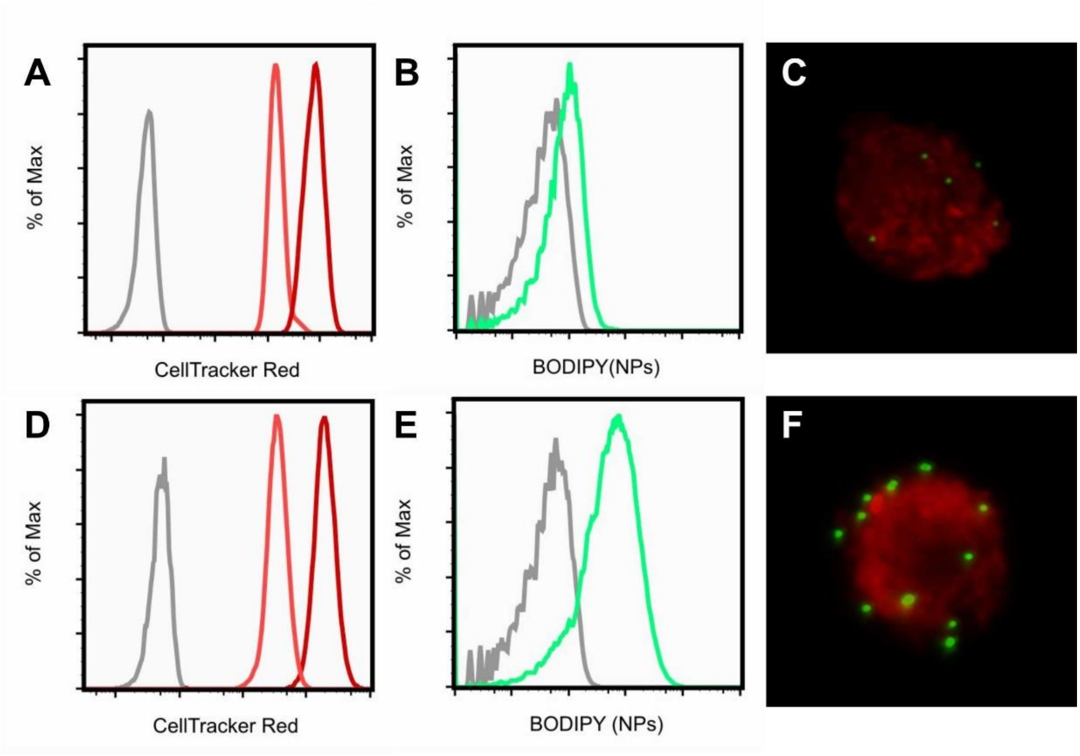

Figure S13. Flow cytometric analysis and representative 3D reconstructions of confocal images of SJL-PLP7 cells labeled with 2.5 μM (A-C) and 1 μM (D-F) CellTracker Red. The CellTracker Red associated channel shows the fluorescence intensity of unmodified control cells on day 3 (grey), cells labeled with CellTracker Red on day 3 (red) and cells labeled with CellTracker Red on day 4 (pale red). The Bodipy associated channel shows the fluorescence intensity of CellTracker Red labeled cells on day 4 before (grey) and after (green) conjugation to nanoparticles. The

reconstructions of confocal images visualize the absolute number of nanoparticles (green) on the surface of CellTracker Red labeled T cells (red). The average amount of nanoparticles per cell (n = 5) was 4.2 ± 2.3 nanoparticles/cell (C) and 12.0 ± 3.7 nanoparticles/cell (F)

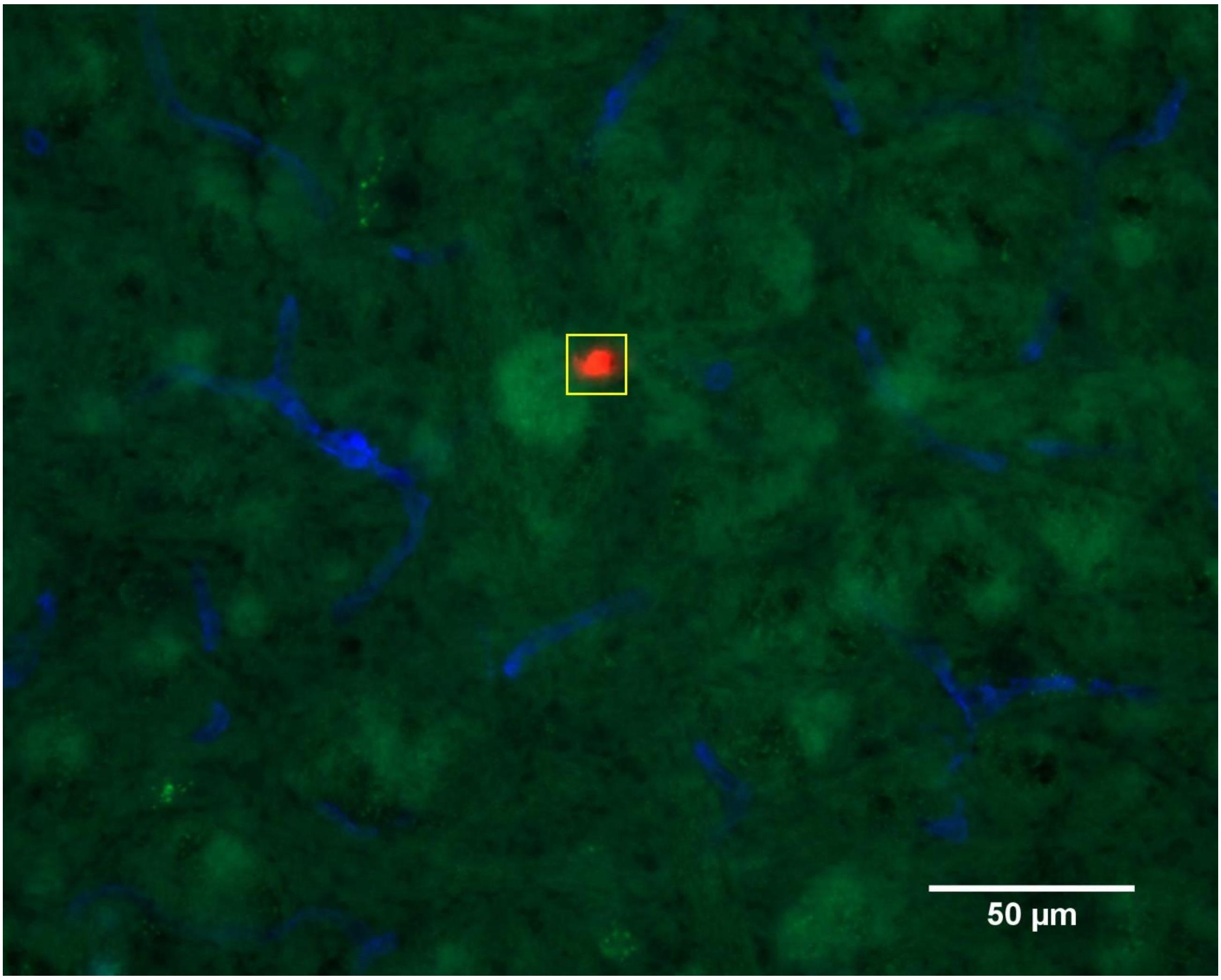

Figure S14: Full-sized Image of **Figure 11A**. Widefield microscopy image of the brain parenchyma of a mouse after injection of CellTracker Red labeled SJL-PLP7 cells. The parenchymal basement membrane was stained for laminin (blue). Cells were labeled with 2.5 µM CellTracker Red. T cells resident in the brain parenchyma are marked by a yellow box.

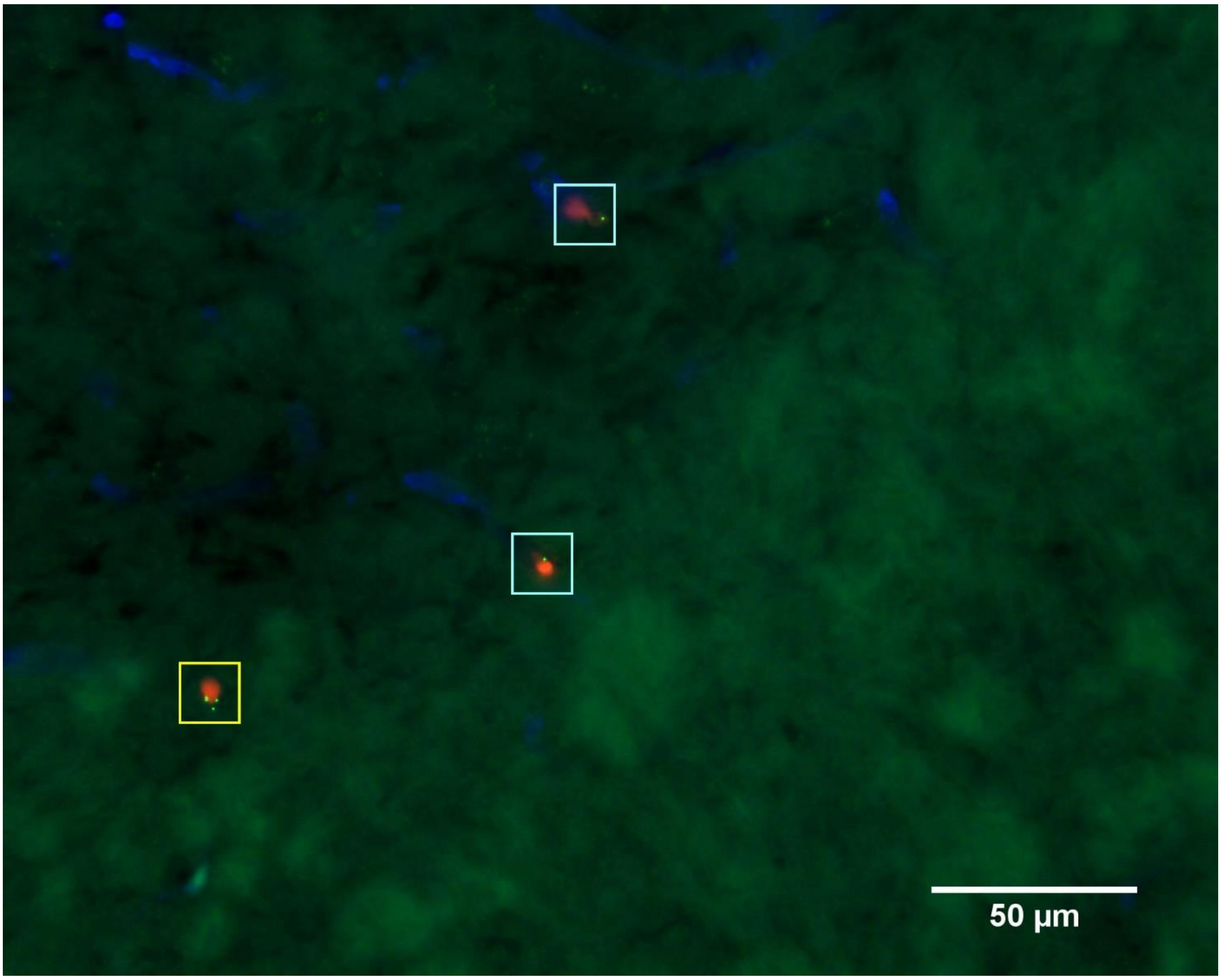

Figure S15: Full-sized Image of **Figure 11B**. Widefield microscopy image of the brain parenchyma of a mouse after injection of CellTracker Red labeled SJL-PLP7 cells conjugated to thiol reactive nanoparticles. The parenchymal basement membrane was stained for laminin (blue). Cells were labeled with 2.5 µM CellTracker Red and modified with thiol reactive nanoparticles (green). T cells resident in the brain parenchyma are indicated by a yellow box. T cells in the brain capillaries are highlighted with a light blue box.

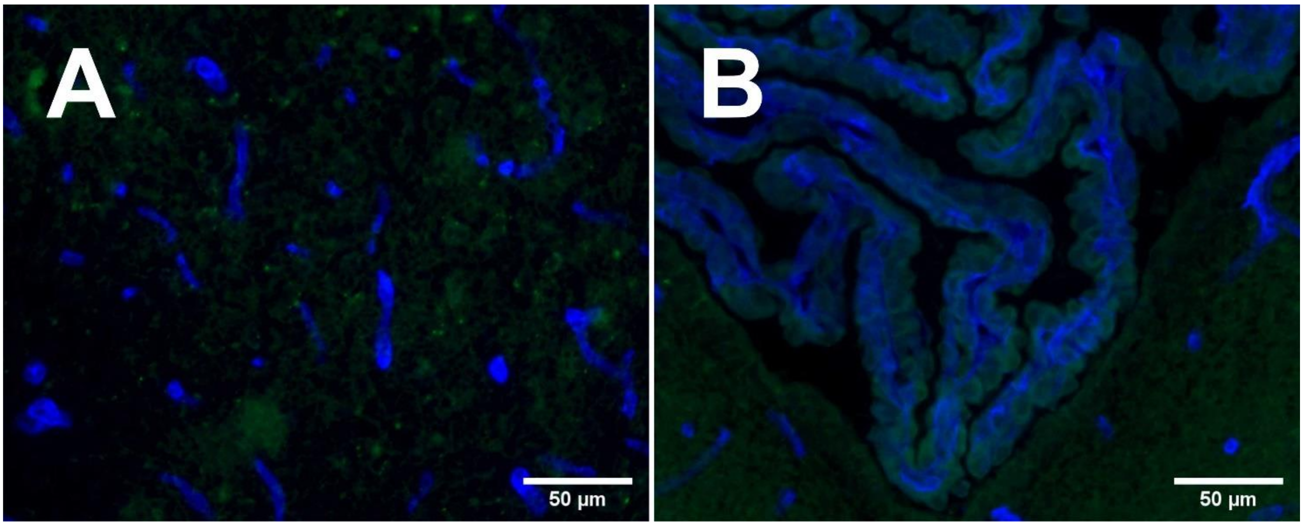

Figure S16: (A) Widefield microscopy image of the brain parenchyma of a mouse after injection of free nanoparticles; (B) Widefield microscopy image of the choroid plexus of a mouse after injection of free nanoparticles. The parenchymal basement membrane was stained for laminin (blue). Cells were labeled with 2.5 µM CellTracker Red (red) and conjugated to thiol reactive fluorospheres (green). Elongated cells that adhere to the endothelium but failed to migrate to the parenchyma are marked with white bars.

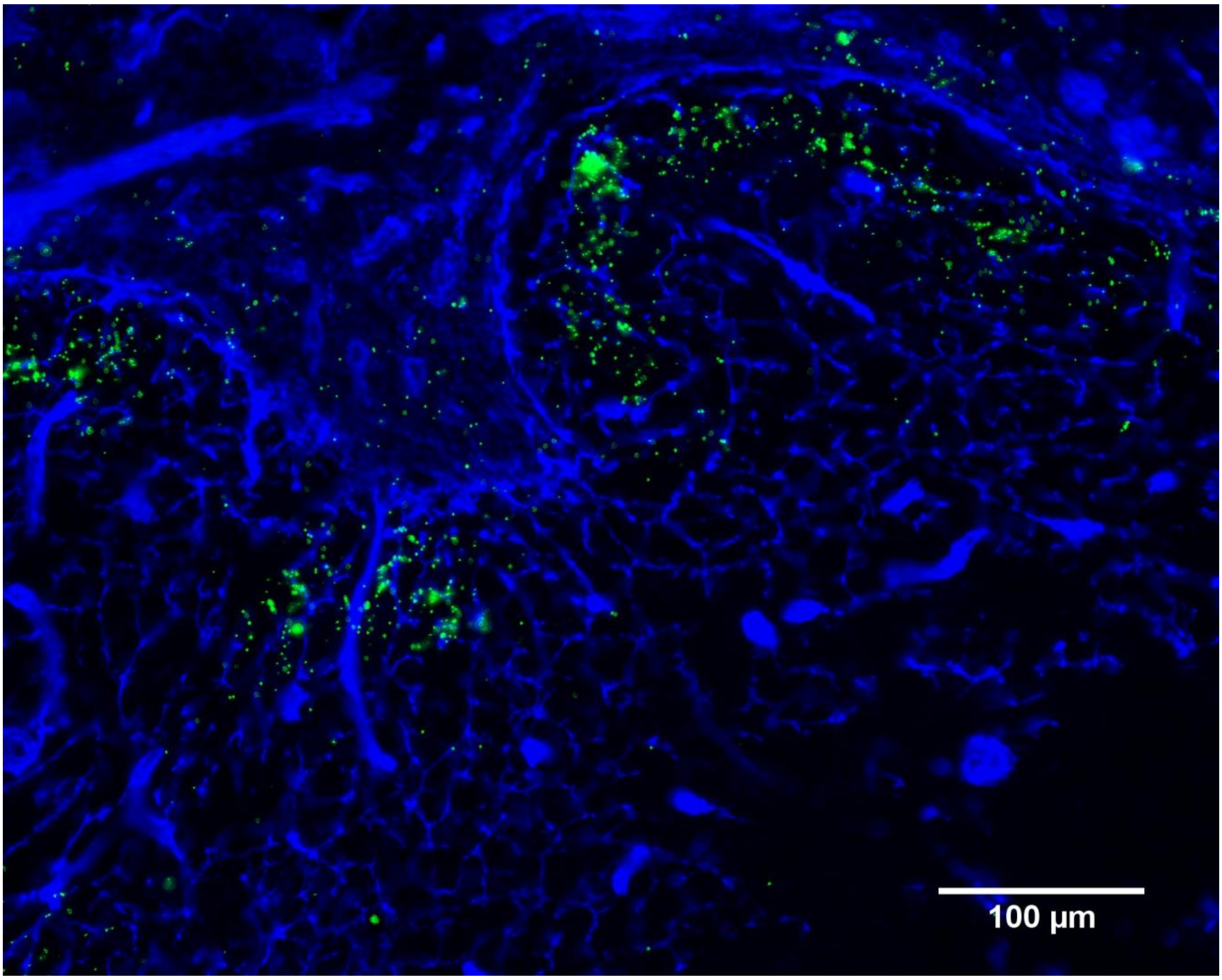

Figure S17: Widefield microscopy image of the spleen of a mouse after injection of free nanoparticles. The basement membranes were stained for laminin (blue). Nanoparticle fluorescence is associated to BODIPY (green).

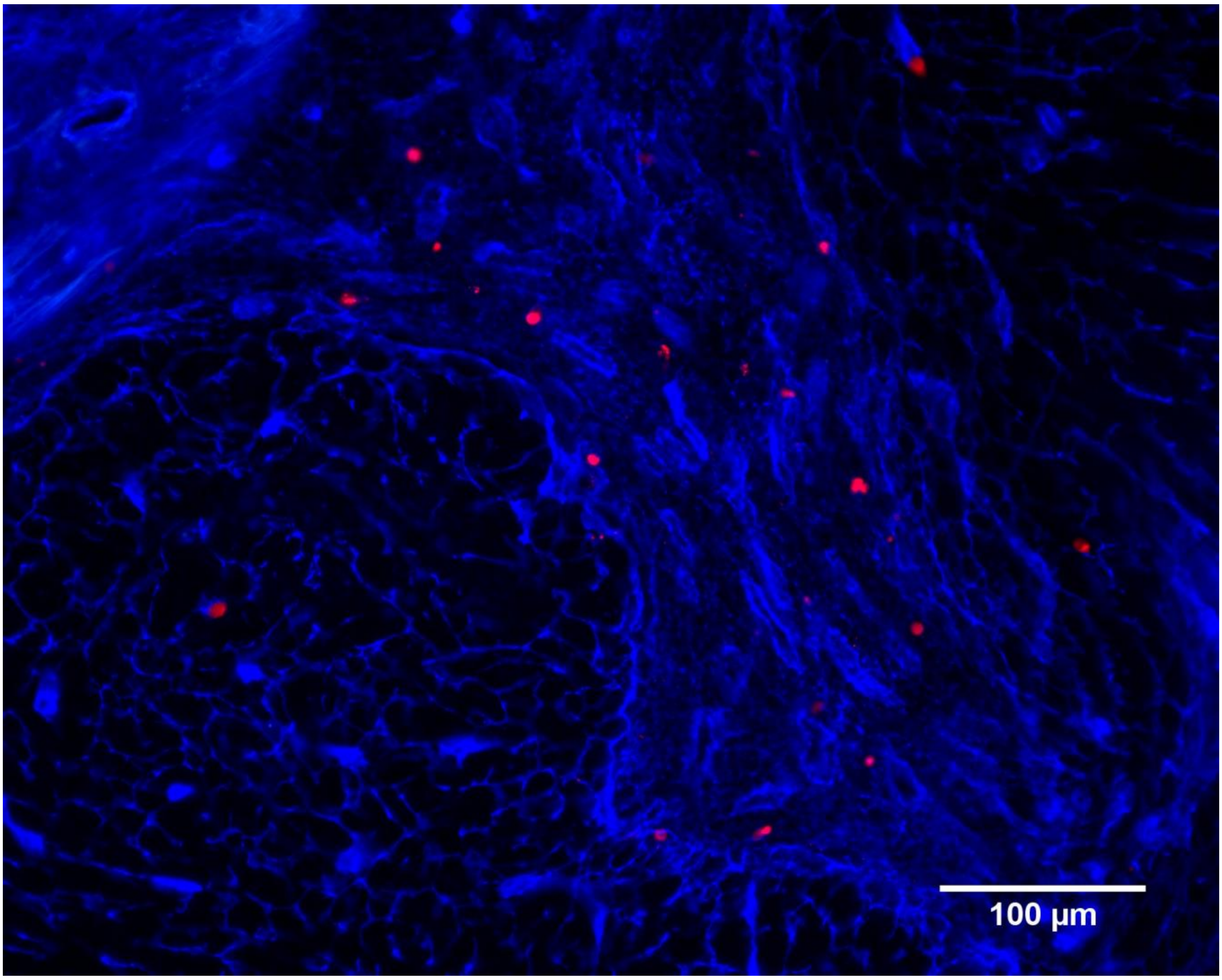

Figure S18: Widefield microscopy image of the spleen of a mouse after injection of CellTracker Red labeled cells. The basement membranes were stained for laminin (blue). Cells were labeled with 2.5 µM CellTracker Red (red).

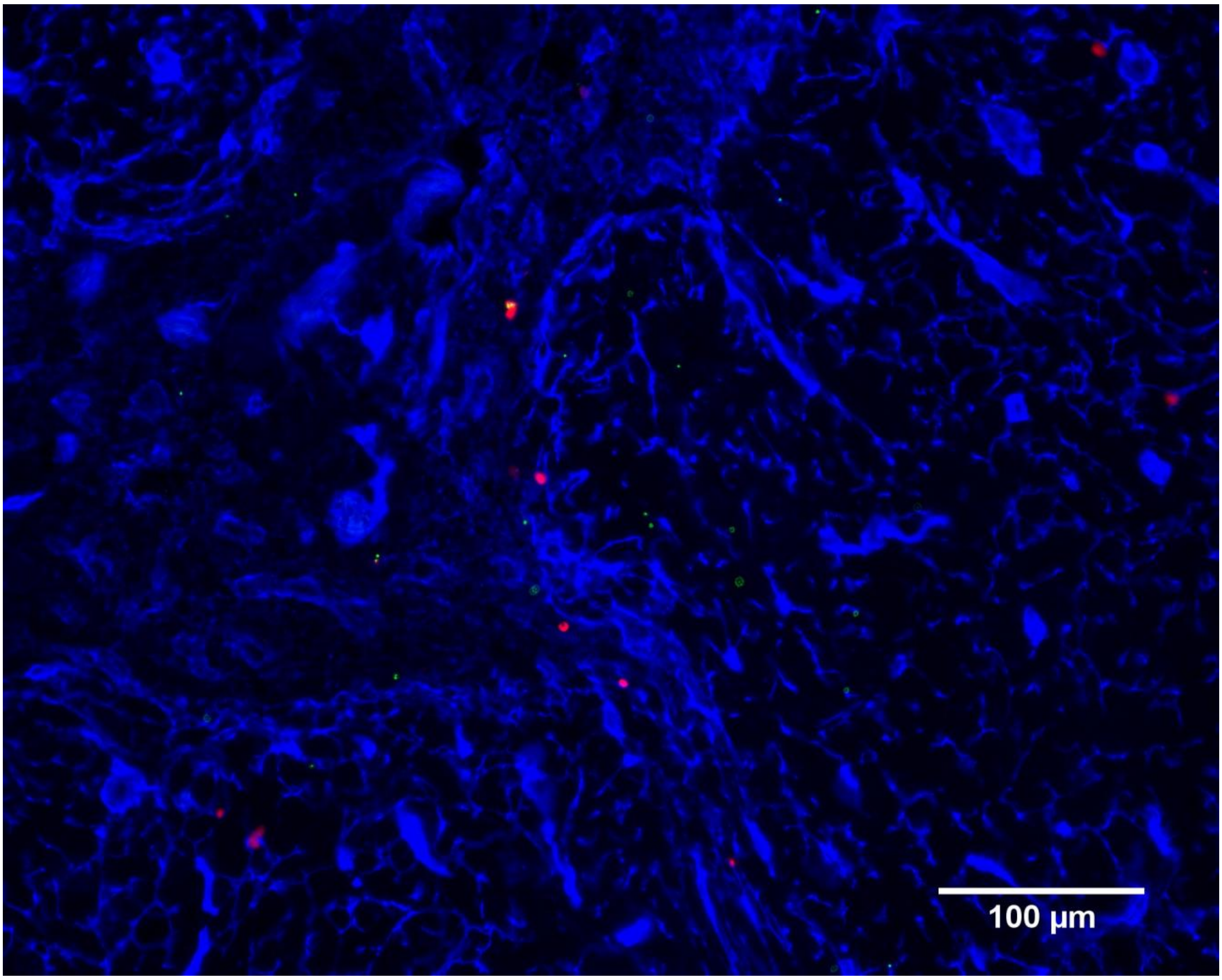

Figure S19: Widefield microscopy image of the spleen of a mouse after injection of CellTracker Red labeled cells conjugated to nanoparticles. The basement membranes were stained for laminin (blue). Cells were labeled with 2.5 µM CellTracker Red (red) and modified with thiol reactive fluorospheres (green).

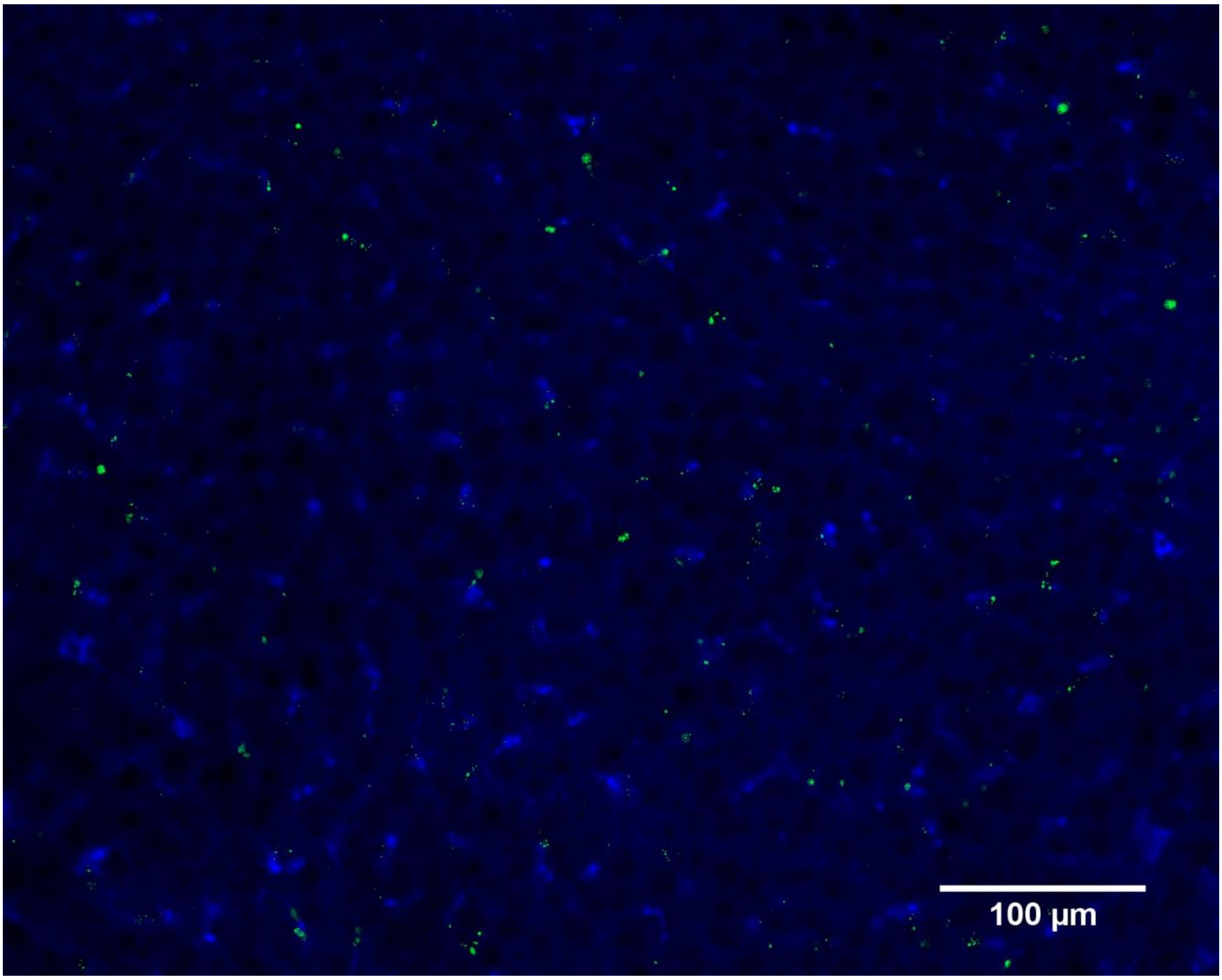

Figure S20: Widefield microscopy image of the liver of a mouse after injection of free nanoparticles. The basement membranes were stained for laminin (blue). Nanoparticle fluorescence is associated to BODIPY (green).

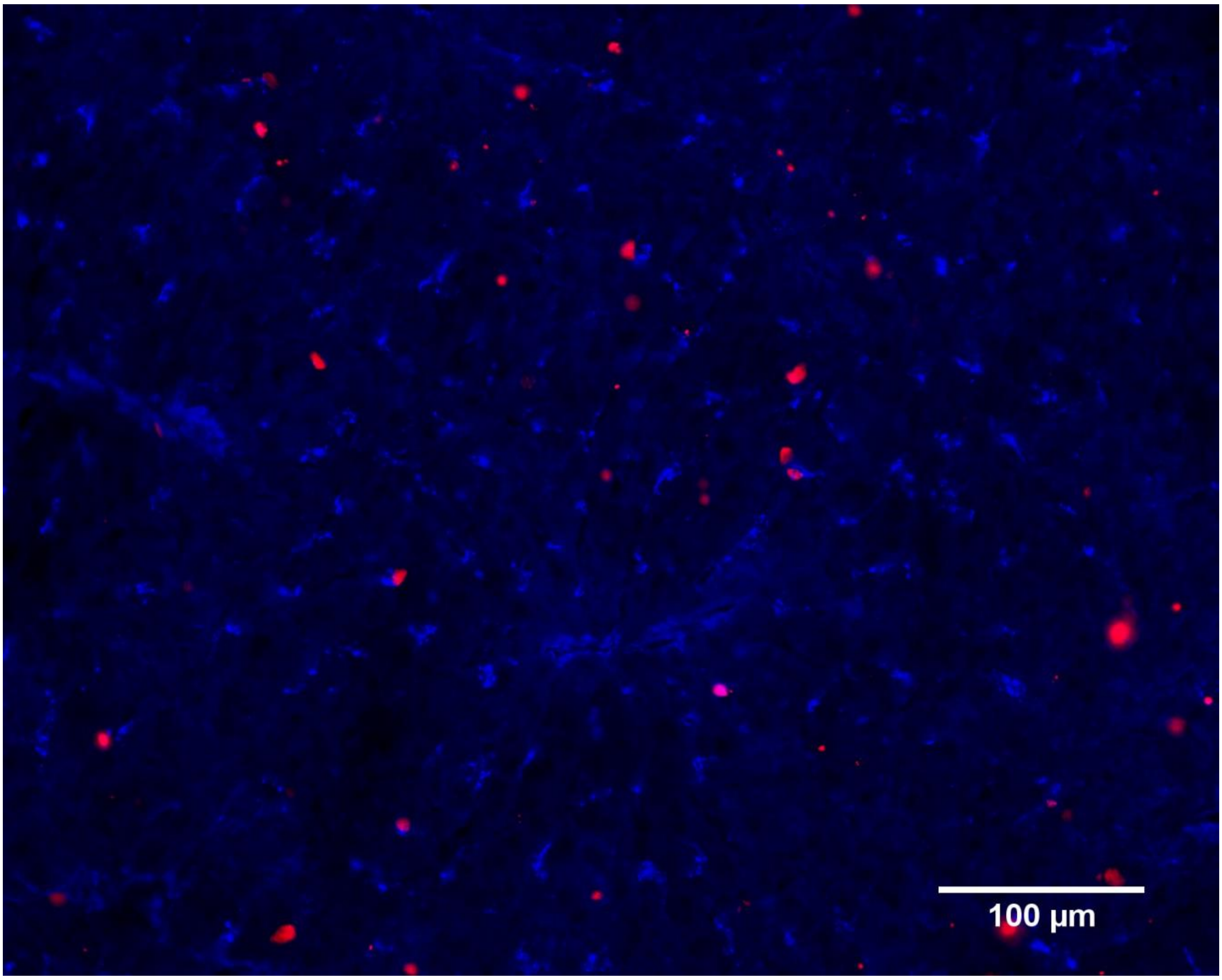

Figure S21: Widefield microscopy image of the liver of a mouse after injection of CellTracker Red labeled cells. The basement membranes were stained for laminin (blue). Cells were labeled with 2.5 µM CellTracker Red (red).

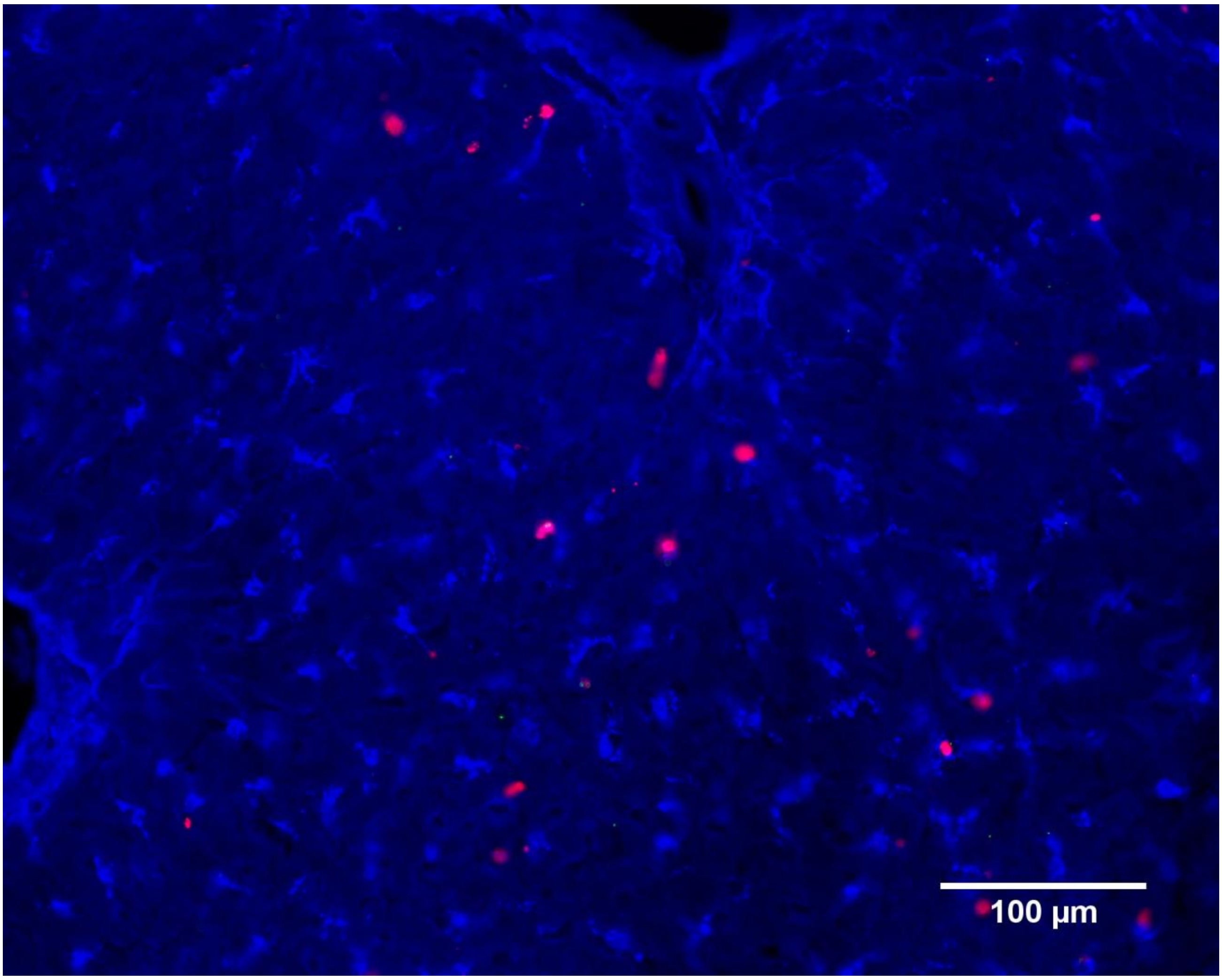

Figure S22: Widefield microscopy image of the liver of a mouse after injection of CellTracker Red labeled cells conjugated to nanoparticles. The basement membranes were stained for laminin (blue). Cells were labeled with 2.5 µM CellTracker Red (red) and modified with thiol reactive fluorospheres (green).
